# Supplementary material for: Aviadenovirus structure: A highly thermostable capsid in the absence of stabilizing proteins
Source: PLoS Pathog. 2025 Oct 9;21(10):e1013553. doi: 10.1371/journal.ppat.1013553 (PMC12517501; doi:10.1371/journal.ppat.1013553)
Supplement: S1 Text — (PDF) [file ppat.1013553.s001.pdf]

Supplementary Text (S1 text) for

Aviadenovirus structure: a highly thermostable capsid in the absence of stabilizing proteins

Marta Pérez-Illana[1,5], Anna Schachner[2,6], Mercedes Hernando-Pérez[1,7], Gabriela N. Condezo[1], Alberto Paradela[1], Marta Martínez[1], Roberto Marabini[3], Michael Hess[2,4], Carmen San Martín[1,*]

[1] Department of Macromolecular Structures, Centro Nacional de Biotecnología (CNB-CSIC). 28049 Madrid, Spain.

[2] Christian Doppler Laboratory for Innovative Poultry Vaccines (IPOV), University of Veterinary Medicine, Vienna, Austria

[3] Escuela Politécnica Superior. Universidad Autónoma de Madrid. 28049 Madrid (Spain)

[4] Clinic for Poultry and Fish Medicine, Department for Farm Animals and Food System Science, University of Veterinary Medicine, Vienna, Austria

[5] Present address: Department of Virology, Aggeu Magalhaes Institute. Oswaldo Cruz Foundation- (FIOCRUZ). PE-50740-465 Recife (Brazil)

[6] Present address: Institute of Medical Genetics, Center for Pathobiochemistry and Genetics, Medical University of Vienna. Währingerstrasse 10, 1090 Vienna, Austria

[7] Present addresses: Department of Materials Physics, Instituto de Ciencia de Materiales Nicolás Cabrera, and Condensed Matter Physics Center (IFIMAC). Universidad Autónoma de Madrid, Ciudad Universitaria de Cantoblanco, 28049, Madrid, Spain.

Short title: Structure of an aviadenovirus, FAdV-C4

*Corresponding author: carmen@cnb.csic.es

Structure of FAdV-C4 strain AG243 lacking pentons

In the process of obtaining a high resolution map of FAdV-C4, a collection of cryo-EM grids prepared under different conditions was screened to select the grid that would contain the optimal ice thickness, particle concentration and intact morphology. One of the grids, prepared from samples of FAdV-C4 strain AG243, was selected and a 3.2 Å resolution map was obtained (**S2 Table, S2a-b Figure**). Unfortunately, although visual inspection of the micrographs did not indicate that the viral particles were damaged, this map showed a capsid completely devoid of pentons (**S2c Figure**). It is known that adenovirus capsid disassembly starts at the pentons [1, 2] and it is relatively common for adenovirus cryo-EM maps to present a low occupancy of pentons [3-5].

Prior works reported that upon freeze-thawing [6], heating or acidification [7], loss of peripheral core materials (presumably protein VI or core protein V) accompanied the release of pentons in HAdVC-5. Apart from the density corresponding to the pentons, our FAdV-C4 pentonless map is also lacking density accounting for connections between capsid and core (**S2c Figure, arrowheads**). These connections arise from the inner hexon cavity, where protein VI is located [8-10]. The loss of internal components at the outermost part of the core is also evidenced by a large dip in the radial average profile of the maps (**S2d Figure, arrowheads**). Another intriguing difference is the loss of densities near the 2-fold symmetry axes (designated as *RD4* in the main text, **Figure 5**) in the pentonless map (**S2c Figure, dots**). The identity and role of this RD is unknown.

Aviadenovirus fibres

The available evidence indicates that, in avian adenoviruses, two fibers bind simultaneously to the same penton base [11, 12], as opposed to human enteric AdVs where each of the two different fiber proteins bind to different vertices [13, 14]. In particular, two fibers attached to a single penton base were observed in negative staining EM images of pentons released from FAdV-C4 strain KR5 virions [12]. This publication reported that the presence of a double fiber in each penton seems to be quite common throughout the aviadenovirus genus.

The question of how two fibers bind to the same penton base is far from trivial. It is not clear whether both fibers interact directly with the penton base, or if one serves as anchoring point to the other; fibers have a thin, β-spiral stalk, which may undergo bending at different points; and the strong signal produced by the icosahedral capsid components

overwhelms that produced by the symmetry mismatched, flexible spikes. As a result, fibres cannot be resolved by cryo-EM single particle averaging when imposing icosahedral symmetry as we have done here, and thus a single blurry stump can be observed, resulting from averaging the proximal end of the two fibres located in each penton vertex (**S2c Figure left, pentagon**). We have not detected weak densities that might correspond to the second fiber positioned nearby. This problem is not exclusive of adenoviruses: a similar phenomenon occurs in the structurally related tectiviruses, and the uncertainties remain even after localized reconstruction was employed to analyze the cryo-EM data [15, 16]. This challenging issue will need to be addressed in the future.

Due to the presence of two trimeric fibres per vertex [11, 12, 17], their interaction with the penton base is expected to be different in aviadenoviruses from other AdVs. A FNPVYPY sequence motif at the fibre N-terminal region has been shown to be involved in attachment to penton base and is conserved in mastadenoviruses [18]. The N-terminal sequences of both FAdV-C4 fibres differ from those in the HAdVs (**S4b Figure, bottom**). While in HAdV-C5 and HAdV-D26 the penton-binding conserved motif (FNPVYPY) is located very close to the protein N-terminus (starting approximately at residue 10), in both FAdV-C4 fibres the equivalent penton-binding peptide (LDLVYPF) is located further downstream, starting only at residue 70 in the short fibre and 60 in the long one (**S4b Figure, dashed rectangle**). A long poly-Gly stretch in the FAdV-C4 short fibre (**S4b Figure, black rectangle**) has been proposed to provide increased flexibility at the region between the N-terminal peptide and the start of the shaft [18].

The N-terminal tails of fibres contact the penton base at the groove between monomers [18]. In our FAdV-C4 map, remnant density on top of the penton base shows a “starfish” shape corresponding to the 5-fold averaged fibre N-termini, similar to density observed in other adenovirus structures. A cylindrical density protruding outwards and corresponding to the 5-fold averaged start of the shafts is also observed (**S4c Figure**). Ten of the eighteen N-terminal amino acids of the HAdV-D26 fibre (containing the FNPVYPY motif) can be docked into the FAdV-C4 remnant density (**S4c Figure**). No additional density that could account for the longer N-terminal tails of the FAdV-C4 fibres (**S4c Figure**), or for the second fibre binding to a different location on the pentamer surface, was observed. In HAdV-D26, it has been proposed that the fibre N-terminal peptide (Ala2-Ala20) folds around the HVL at the periphery of the penton base pentamer **(S4d Figure, left**) [19]. If we overlap the HAdV-D26 fibre N-terminal peptide with the FAdV-C4 penton base structure, we observe that the HVL, VL and VL’ loops, which fold

back towards the main body of the pentamer instead of spreading out into the solvent, could provide additional interactions with the double fibre N-terminal tails and contribute to clasp them to the capsid (**S4d Figure, right**). Alternatively, this conformational difference could allow the accommodation of the extra-long N-terminal region preceding the penton binding motif (**S4c Figure**). Further studies using symmetry relaxation and localized reconstruction would be needed to solve the mode of binding of the two trimeric fibres to the pentameric pentons.

References

1. Greber UF, Willetts M, Webster P, Helenius A. Stepwise dismantling of adenovirus 2 during entry into cells. Cell. 1993;75(3):477-86. PubMed PMID: 8221887.
2. Ortega-Esteban A, Pérez-Berná AJ, Menéndez-Conejero R, Flint SJ, San Martín C, de Pablo PJ. Monitoring dynamics of human adenovirus disassembly induced by mechanical fatigue. Scientific reports. 2013;3:art. no. 1434. doi: 10.038/srep01434. Epub 2013/03/15. doi: 10.1038/srep01434. PubMed PMID: 23486377; PubMed Central PMCID: PMC3595926.
3. Marsh MP, Campos SK, Baker ML, Chen CY, Chiu W, Barry MA. CryoEM of Protein IX-Modified Adenoviruses Suggests a New Position for the C-terminus of Protein IX. J Virol. 2006;80:11881-6. PubMed PMID: 16987967.
4. Cheng L, Huang X, Li X, Xiong W, Sun W, Yang C, et al. Cryo-EM structures of two bovine adenovirus type 3 intermediates. Virology. 2014;450-451:174-81. doi: 10.1016/j.virol.2013.12.012. PubMed PMID: 24503080.
5. Marabini R, Condezo GN, Krupovic M, Menéndez-Conejero R, Gómez-Blanco J, San Martín C. Near-atomic structure of an atadenovirus reveals a conserved capsid-binding motif and intergenera variations in cementing proteins. Sci Adv. 2021;7(14). Epub 2021/04/02. doi: 10.1126/sciadv.abe6008. PubMed PMID: 33789897; PubMed Central PMCID: PMCPMC8011978.
6. Martinez R, Schellenberger P, Vasishtan D, Aknin C, Austin S, Dacheux D, et al. The amphipathic helix of adenovirus capsid protein VI contributes to penton release and postentry sorting. J Virol. 2015;89(4):2121-35. doi: 10.1128/JVI.02257-14. PubMed PMID: 25473051; PubMed Central PMCID: PMCPMC4338868.
7. Pérez-Berná AJ, Ortega-Esteban A, Menéndez-Conejero R, Winkler DC, Menéndez M, Steven AC, et al. The role of capsid maturation on adenovirus priming for sequential uncoating. The Journal of biological chemistry. 2012;287(37):31582-95.

Epub 2012/07/14. doi: 10.1074/jbc.M112.389957. PubMed PMID: 22791715; PubMed Central PMCID: PMC3438990.

8. Dai X, Wu L, Sun R, Zhou ZH. Atomic Structures of Minor Proteins VI and VII in Human Adenovirus. J Virol. 2017;91(24). doi: 10.1128/JVI.00850-17. PubMed PMID: 28978703; PubMed Central PMCID: PMCPMC5709574.

9. Hernando-Pérez M, Martín-González N, Pérez-Illana M, Suomalainen M, Condezo GN, Ostapchuk P, et al. Dynamic competition for hexon binding between core protein VII and lytic protein VI promotes adenovirus maturation and entry. Proc Natl Acad Sci U S A. 2020;117(24):13699-707. Epub 2020/05/30. doi: 10.1073/pnas.1920896117. PubMed PMID: 32467158; PubMed Central PMCID: PMCPMC7306765.

10. Yu X, Mullen T-M, Abrishami V, Huiskonen JT, Nemerow GR, Reddy VS. Structure of a Cell Entry Defective Human Adenovirus Provides Insights into Precursor Proteins and Capsid Maturation. Journal of Molecular Biology. 2022;434(2):167350. doi: https://doi.org/10.1016/j.jmb.2021.167350.

11. Hess M, Cuzange A, Ruigrok RWH, Chroboczek J, Jacrot B. The avian adenovirus penton: two fibres and one base. J Mol Biol. 1995;252(4):379-85. PubMed PMID: 7563058.

12. Gelderblom H, Maichle-Lauppe I. The fibers of fowl adenoviruses. Arch Virol. 1982;72(4):289-98. Epub 1982/01/01. doi: 10.1007/BF01315225. PubMed PMID: 6287974.

13. Favier AL, Schoehn G, Jaquinod M, Harsi C, Chroboczek J. Structural studies of human enteric adenovirus type 41. Virology. 2002;293(1):75-85. Epub 2002/02/21. doi: 10.1006/viro.2001.1235. PubMed PMID: 11853401.

14. Kidd AH, Chroboczek J, Cusack S, Ruigrok RWH. Adenovirus type 40 virions contain two distinct fibers. Virology. 1993;192(1):73-84. PubMed PMID: 8517033.

15. Reddy HK, Carroni M, Hajdu J, Svenda M. Electron cryo-microscopy of bacteriophage PR772 reveals the elusive vertex complex and the capsid architecture. Elife. 2019;8. doi: 10.7554/eLife.48496. PubMed PMID: 31513011; PubMed Central PMCID: PMCPMC6750898.

16. Huiskonen J, Manole V, Butcher S. Tale of two spikes in bacteriophage PRD1. Proc Natl Acad Sci U S A. 2007;104(16):6666-71.

17. Benkö M, Aoki K, Arnberg N, Davison AJ, Echavarria M, Hess M, et al. ICTV Virus Taxonomy Profile: Adenoviridae 2022. J Gen Virol. 2022;103(3). Epub

2022/03/10. doi: 10.1099/jgv.0.001721. PubMed PMID: 35262477; PubMed Central PMCID: PMCPMC9176265.

18. Zubieta C, Schoehn G, Chroboczek J, Cusack S. The structure of the human adenovirus 2 penton. Mol Cell. 2005;17(1):121-35. PubMed PMID: 15629723.

19. Yu X, Veesler D, Campbell MG, Barry ME, Asturias FJ, Barry MA, et al. Cryo-EM structure of human adenovirus D26 reveals the conservation of structural organization among human adenoviruses. Sci Adv. 2017;3(5):e1602670. Epub 2017/05/17. doi: 10.1126/sciadv.1602670. PubMed PMID: 28508067; PubMed Central PMCID: PMCPMC5425241.
