# Supplementary material for: Aviadenovirus structure: A highly thermostable capsid in the absence of stabilizing proteins
Source: PLoS Pathog. 2025 Oct 9;21(10):e1013553. doi: 10.1371/journal.ppat.1013553 (PMC12517501; doi:10.1371/journal.ppat.1013553)
Supplement: S1 Table — (PDF) [file ppat.1013553.s002.pdf]

S1 Table. FAdV-C4 proteins identified by LC-MS/MS

Protein	Mass of immature protein (Da)	MASCOT Score	Total number of peptide (number of significant) matches	Sequences	Sum of peptide area (x10^6)
hexon	106267	2203	52 (52)	40 (40)	86.41
pX (pre-μ)[a]	18649	1006	27 (27)	22 (22)	61.07
pVIII[a]	26956	749	18 (18)	16 (16)	8.69
pIIIa[a]	65275	649	16 (16)	16 (16)	2.91
penton base	57883	562	13 (13)	13 (13)	10.41
pVI[a]	24302	428	12 (12)	9 (9)	1.41
pVII[a]	9031	417	16 (16)	12 (12)	10.54
L1 52/55k[a]	44532	244	9 (9)	8 (8)	1.09
fibre-1	45089	238	5 (5)	5 (5)	0.98
fibre-2	50066	96	3 (3)	3 (3)	0.78
IVa2	45810	56	2 (2)	2 (2)	0.09
AVP	24309	55	2 (2)	2 (2)	0.87
pTP[a]	70648	35	1 (1)	1 (1)	0.12

[a] Proteins cleaved by AVP during maturation (see **S1 Figure**)
