# Supplementary material for: Aviadenovirus structure: A highly thermostable capsid in the absence of stabilizing proteins
Source: PLoS Pathog. 2025 Oct 9;21(10):e1013553. doi: 10.1371/journal.ppat.1013553 (PMC12517501; doi:10.1371/journal.ppat.1013553)
Supplement: S2 Table — (PDF) [file ppat.1013553.s003.pdf]

S2 Table. Cryo-EM data collection, image processing, reconstruction and refinement for the two FAdV-C4 specimens used

**Data collection**	**FAdV-C4 KR5**	**FAdV-C4 AG234**
Microscope	Titan Krios	Titan Krios
Camera	Falcon III (linear)	Falcon III (linear)
Voltage	300 kV	300 kV
Magnification	97,902	95,890
Nominal pixel size	1.43 Å/px	1.46 Å/px
Dose rate	35 $e^{-}/Å^{2}.s$	41$e^{-}/Å^{2}.s$
Exposure time	1.1 s	0.99 s
Cumulative electron dose	38.6 e-/$Å^2$	40.5 e-/$Å^2$
Number of frames	39	39
Defocus range	-1 to -2.5 µm	-1 to -2.5 µm
Micrographs collected	4,306	4,796
Acquisition software	EPU	EPU
**Image processing**		
Frame alignment software	Motioncor2 Scipion	Motioncor2, Scipion
CTF estimation software	gCTF, Scipion	gCTF, Scipion
Particle picking software	XMIPP, Scipion	XMIPP, Scipion
Micrographs used	3,769	4,733
Particles picked	13,587	40,383
Particles after screening	10,919	40,345
Particles after 2D classes	10,877	40,169
**Reconstruction**		
Software	RELION, Scipion	RELION, Scipion
Particles included	9,466	32,425
Symmetry imposed	Icosahedral	Icosahedral
Rotational accuracy	0.05 degrees	0.05 degrees
Translational accuracy	0.11 pixels	0.1 pixels
B-factor applied	-196 $Å^2$	-211 $Å^2$
Final resolution (gold standard FSC=0.143)	3.3 $Å^2$	3.2 $Å^2$
Experimental pixel size	1.375 Å/px	1.375 Å/px
