# Supplementary material for: Aviadenovirus structure: A highly thermostable capsid in the absence of stabilizing proteins
Source: PLoS Pathog. 2025 Oct 9;21(10):e1013553. doi: 10.1371/journal.ppat.1013553 (PMC12517501; doi:10.1371/journal.ppat.1013553)
Supplement: S3 Table — (PDF) [file ppat.1013553.s004.pdf]

S3 Table. Modelling and validation statistics for FAdV-C4 KR5 [a]

**Model building, refinement and validation**	
Software	Scipion, Xmipp, UCSF Chimera, UCSF ChimeraX, Coot, Phenix
Chains	16
Atoms	97638 (Hydrogens: 0)
Residues	Protein: 12237 Nucleotide: 0
Water	**0**
Ligands	**0**
**Bonds (RMSD); outliers >4σ**	
Length (Å)	0.005; 0
Angles (°)	1.03; 8
MolProbity score	2.21
Clash score	16.6
**Ramachandran plot (%)**	
Outliers	0.04
Allowed	8.02
Favored	91.94
**Rama-Z (Ramachandran plot Z-score, RMSD)**	
whole	(N= 12201) -2.02 (0.07)
helix	(N= 1835) -1.18 (0.11)
sheet	(N= 2563) -1.05 (0.11)
loop	(N= 780.3) -1.53 (0.07)
**Rotamer outliers (%)**	0.04
**Cβ outliers (%)**	0
**Peptide plane**	
Cis proline/general	0.0/0.0
Twisted proline/general	0.3/0.0
**CaBLAM outliers (%)**	5.33
**ADP (B-factors)**	
Iso/Aniso	97638/0
min/max/mean	
Protein	24.96/94.00/41.69
**Model vs. Data**	
CC (mask)	0.85
CC (box)	0.49
CC (peaks)	0.29
CC (volume)	0.81

[a]Obtained from Phenix v19 cryo-EM comprehensive validation. No model was built for FAdV-C4 AG234
