# Supplementary material for: Aviadenovirus structure: A highly thermostable capsid in the absence of stabilizing proteins
Source: PLoS Pathog. 2025 Oct 9;21(10):e1013553. doi: 10.1371/journal.ppat.1013553 (PMC12517501; doi:10.1371/journal.ppat.1013553)
Supplement: S4 Table — (PDF) [file ppat.1013553.s005.pdf]

S4 Table. FAdV-C4 proteins traced in the model

Protein	Length (amino acids)	Copy number in AU	Chain ID	Residues traced	Not traced	Number traced
**hexon**	937	12	A	11-937	1-10	927
			B	2-935	1; 936-937	934
			C	4-937	1-3	934
			D	10-937	1-9	928
			E	11-937	1-10	927
			F	2-936	1; 937	935
			G	8-937	1-7	930
			H	11-935	1-10; 936-937	925
			I	11-937	1-10	927
			J	11-936	1-10; 937	926
			K	2-937	1	936
			L	11-937	1-10	927
**penton base**	525	1	M	54-524	1-53; 525	471
**IIIa**	590	1	N	16-259	1-16; 259-590	244
**IIIa APD***				305-384		80
**VIII**	247	2	O	1-115; 174-241	116-173; 242-247	183
			P	1-115; 174-241	116-173; 242-247	183

*Tentative assignment (**S6c Figure**).
