# Supplementary material for: Aviadenovirus structure: A highly thermostable capsid in the absence of stabilizing proteins
Source: PLoS Pathog. 2025 Oct 9;21(10):e1013553. doi: 10.1371/journal.ppat.1013553 (PMC12517501; doi:10.1371/journal.ppat.1013553)
Supplement: S5 Table — (PDF) [file ppat.1013553.s006.pdf]

S5 Table. Comparison between HAdV-C5 and FAdV-C4 virion proteins

	Length (aa)		Molecular weight (kDa)		Isoelectric Point		Charge at pH 7		% sequence homology	
	HAdV-C5	FAdV-C4	HAdV-C5	FAdV-C4	HAdV-C5	FAdV-C4	HAdV-C5	FAdV-C4	Pairwise identity	Pairwise Positive
**hexon**[a]	952	937	108.007	106.049	4.94	4.88	-23.94	-19.22	44.5	58.1
**penton base**[a]	571	525	63.293	57.406	5.15	4.72	-12.33	-10.29	37.9	50.5
**IIIa**[a]	585	590	65.253	65.259	5.71	6.66	-7.05	-0.64	24.4	37.7
**VIII**[a]	227	247	24.687	26.859	9.24	6.15	2.41	-1.02	22.5	36.8
**VI**[b]	250	227	26.996	24.260	10.42	11.02	8.06	11.35	19.0	34.4
**VII**[b]	198	77	21.992	9.037	12.45	13.48	40.31	24.91	14.5	19.0
**V**	368	absent	41.447	Absent	10.75	absent	27.34	absent	--	--
**µ**	80	179	8.846	18.660	12.98	12.21	17.43	27.91	14.8	18.6
**52/55k**	415	399	47.060	44.389	5.47	5.08	-10.01	-10.47	24.0	37.6
**fibre 1**	581	433	61.585	45.059	6.01	4.53	-3.51	-7.95	--	--
**fibre 2**	absent	479	absent	49.812	absent	4.07	absent	-20.01	--	--
**AVP**	204	209	23.068	24.039	8.21	9.29	5.17	7.27	36.7	63.8
**TP**	671	602	76.500	70.407	5.91	6.75	-7.3	-1.09	27.9	43.6
**IVa2**	449	394	50.887	45.383	8.69	7.41	8.34	1.58	28.0	43.3
**IX**[b]	140	absent	14.458	absent	6.45	absent	-0.09	absent	--	--

[a] Proteins traced (totally or in part) in both HAdV-C5 and FAdV-C4

[b] Proteins traced only in HAdV-C5 model (Dai *et al.*, 2017)

AVP: adenovirus protease. TP: Terminal protein. Hexon, penton base, VII, 52/55k, TP and IVa2 are shorter in FAdV-C4 compared to HAdV-C5, whereas IIIa, VIII, µ and AVP are longer. The FAdV-C4 virion has two shorter and more acidic fibres compared to the single fibre in HAdV-C5. Proteins IX and V contribute outer negative and inner positive charges in HAdV-C5, but they are not present in the FAdV-C4 virions.
