# Supplementary material for: Aviadenovirus structure: A highly thermostable capsid in the absence of stabilizing proteins
Source: PLoS Pathog. 2025 Oct 9;21(10):e1013553. doi: 10.1371/journal.ppat.1013553 (PMC12517501; doi:10.1371/journal.ppat.1013553)
Supplement: S6 Table — (PDF) [file ppat.1013553.s007.pdf]

S6 Table. Regions of hexon with differences between HAdV-C5 and FAdV-C4[a].

Different region[b]	Amino acids in HAdV-C5	Amino acids in FAdV-C4	Observation
diff 1	absent	M1-T5	N-term
diff 2	W135-V168	S140-A150	HVR 1
diff 3	G176-K198	Y158-S176	HVR 2[c]
diff 4	Y212-A220	P190-L202	HVR 3
diff 5	K226-W227	K207	Surface exposed
diff 6	I249-Q261	L229-T230	HVR 4
diff 7	S268-V284	M237-G246	HVR 5
diff 8	P304-G316	P267-N275	HVR 6[c]
diff 9	G419-TR453	A379-Y417	HVR 7
diff 10	N491-K493	D457-T459	Big insertion neighborhood
diff 11	~V749	V714-A715	Pincer
diff 12	K810-Y811	A776	Pincer
diff 13	Y787	Y756	Inside hexon cavity
diff 14	~L820	S785-S798	Big insertion
diff 15	~W814	S813-V816	Big insertion neighborhood
diff 16	D857	Q840-G842	Pincer
diff 17	T952	absent	C-term

[a] Two criterions were used to determine these regions: residues exceeding an RMSD cutoff of 5 Å, or insertions/deletions in either of the two sequences.

[b] Different regions sorted by sequence order.

[c] *diff3* and *diff8* extend beyond the previously reported boundaries of HVR2 and HVR6 in HAdV-C5 [1].

Reference

1. Rux JJ, Kuser PR, Burnett RM. Structural and phylogenetic analysis of adenovirus hexons by use of high-resolution x-ray crystallographic, molecular modeling, and sequence-based methods. J Virol. 2003;77(17):9553-66. Epub 2003/08/14. doi: 10.1128/jvi.77.17.9553-9566.2003. PubMed PMID: 12915569; PubMed Central PMCID: PMCPMC187380.
