# Supplementary material for: Aviadenovirus structure: A highly thermostable capsid in the absence of stabilizing proteins
Source: PLoS Pathog. 2025 Oct 9;21(10):e1013553. doi: 10.1371/journal.ppat.1013553 (PMC12517501; doi:10.1371/journal.ppat.1013553)
Supplement: S7 Table — (PDF) [file ppat.1013553.s008.pdf]

S7 Table. Intra-monomer interactions established by hexon *big insertion*

**Amino acids in *big insertion***	**Interacting amino acids**
Ser785	Gln799
Tyr786	Tyr449, Leu475, Val478, Gln493
Pro788	Arg448, Lys450, Leu475
Asn792	Ser454, Phe456
Ser793	Ser454
Gly794	Pro268, Ser452, Ser454, Pro815
Glu795	Pro268, Ser813, Trp814
Gln796	Phe451, Leu475, Gln799, Ser813
Pro797	Ser813
