# Supplementary material for: Aviadenovirus structure: A highly thermostable capsid in the absence of stabilizing proteins
Source: PLoS Pathog. 2025 Oct 9;21(10):e1013553. doi: 10.1371/journal.ppat.1013553 (PMC12517501; doi:10.1371/journal.ppat.1013553)
Supplement: S8 Table — (PDF) [file ppat.1013553.s009.pdf]

**S8 Table.** Different regions of penton base in HAdV-C5 and FAdV-C4.

| Different region[a] | Amino acids in HAdV-C5 | Amino acids in FAdV-C4 | Observation |
|---|---|---|---|
| *diff 1* | M1-L36 | M1-M53 | At the start of the N-terminal arm, non-traced[b] |
| *diff 2* | S76-S81 | T93-D97 | Located at the base, interaction with hexon. Specificity in hexon-penton interaction? |
| *diff 3* | P153-Q158 | P165-V181 | VL: longer in FAdV-C4 |
| *diff 4* | A294-D397 | N318-D328 | HVL: Shorter and lacks RGD in FAdV-C4. If located as in HAdV-C5, it would clash against the VL |
| *diff 5* | ~D397 | D347-K352 | Insertion in FAdV-C4. Given its length, proposed to be another variable loop (VL') |
| *diff 6* | G413-Q416 | P367 | Surface exposed |
| *diff 7* | S458 | N407 | Located at the base, interaction with hexon. Specificity in hexon-penton interactions? |
| *diff 8* | T493 | T446 | Fibre rearrangement region |

[a]Different regions sorted by sequence order.

[b]*diff1* is defined by sequence comparison and not by the RMSD> 5 Å criterion
