# Supplementary material for: Aviadenovirus structure: A highly thermostable capsid in the absence of stabilizing proteins
Source: PLoS Pathog. 2025 Oct 9;21(10):e1013553. doi: 10.1371/journal.ppat.1013553 (PMC12517501; doi:10.1371/journal.ppat.1013553)
Supplement: S9 Table — (PDF) [file ppat.1013553.s010.pdf]

S9 Table. Comparison of intra-monomer interactions established by penton base variable loop (VL) in HAdV-C5 and FAdV-C4

**HAdV-C5**		**FAdV-C4**	
**Amino acids in VL**	**Interacting amino acids**	**Amino acids in VL**	**Interacting amino acids**
Pro153<br>Gln158	Leu152, Val159<br>Val 159	Pro165	Asp 164, Asn223
		Pro166	Arg163, Gln225
		Pro171	His335, Gly339
		Pro172	Leu334, Ser341
		Ser173	Leu334
		Val175	Pro332, Leu334,Val344,Tyr34, Pro353
		Gly176	Val344, Ile345
		Tyr179	Leu266, Pro267, Asn343, Ile345
		Val181	Met160, Arg163, Gly183, Ala184
