# Supplementary material for: Aviadenovirus structure: A highly thermostable capsid in the absence of stabilizing proteins
Source: PLoS Pathog. 2025 Oct 9;21(10):e1013553. doi: 10.1371/journal.ppat.1013553 (PMC12517501; doi:10.1371/journal.ppat.1013553)
Supplement: S10 Table — (PDF) [file ppat.1013553.s011.pdf]

S10 Table. Regions of protein VIII with RMSD> 5 Å between HAdV-C5 and FAdV-C4.

Different region[a]	Amino acids in HAdV-C5	Amino acids in FAdV-C4	Observations
diff 1	S2-G4	M1-A6	Longer N-terminal in FAdV-C4 (interactions)
diff 2	A59-N103	W60-V107	Boundaries of α2' and α2'' in FAdV-C4
diff 3	G158-Q172	P174-V187	β4 in HAdV-C5/ α2''' in FAdV-C4 (neck)
diff4	~S222	V233-G236	Insertion in FAdV-C4 near C-terminus (interactions)
diff 5	Y226-D227	E242-G247	Flexible and non-traced in FAdV-C4 (interactions)

[a]Different regions sorted by sequence order.
