# Supplementary material for: Aviadenovirus structure: A highly thermostable capsid in the absence of stabilizing proteins
Source: PLoS Pathog. 2025 Oct 9;21(10):e1013553. doi: 10.1371/journal.ppat.1013553 (PMC12517501; doi:10.1371/journal.ppat.1013553)
Supplement: S11 Table — (PDF) [file ppat.1013553.s012.pdf]

S11 Table. Different regions of protein IIIa in HAdV-C5 and FAdV-C4.

Different region[a]	Amino acids in HAdV-C5	Amino acids in FAdV-C4	Observation
diff 1	D4-N41	A 19-A31	Shorter in FAdV-C4 and flexible
diff 2	R128-G135	V 117-R 126	End of the connecting helix and loop to VIII-binding domain, causing its drastic orientation change. FAdV-C4 has a 2 amino acid insertion
diff 3[b]	R193-Q194	M184-G186	Handle
diff 4[b]	A 216-S225	W208-G217	Not traced in HAdV-C5 but forms alpha helix in FAdV-C4
diff 5[b]	A 268-L301	…	Not ordered in FAdV-C4

[a]Different regions sorted by sequence order.

[b]*diff 3, 4 and 5* were defined by comparison of the structures after superposition of the VIII-binding domains
