# Supplementary material for: Aviadenovirus structure: A highly thermostable capsid in the absence of stabilizing proteins
Source: PLoS Pathog. 2025 Oct 9;21(10):e1013553. doi: 10.1371/journal.ppat.1013553 (PMC12517501; doi:10.1371/journal.ppat.1013553)
Supplement: S12 Table — (PDF) [file ppat.1013553.s013.pdf]

S12 Table. Interactions between hexons in the same facet (ST interfaces). For interface nomenclature, see **S9 Figure.**

ST1: H2—H1

E	Ile76	Ile76	A
E	Gln77	Tyr74, Ile76	A
E	Asp79	Arg72, Tyr74	A
E	Arg86	Tyr74	A
E	Glu311	Asn92, Arg919	A
E	Arg312	Gly94,Asp95,Arg919	A
E	Ser313	Asp95	A
E	Gly314	Gly94, Trp97	A
E	Met315	Lys65-Gln67,Trp97,His589	A
E	Glu547	Tyr74	A
E	Ala936	Glu64	A
E	Val937	Thr62, Glu64	A
E	Thr81-Thr83	Thr718	C
E	Arg86	Glu719	C
E	Gly302	Pro705	C
E	Val303	Leu703, Thr704,Phe721	C
E	Val319	Asp700	C
E	Pro322	Asn706	C
E	Lys615	Ser693	C
E	Pro633	Ser886	C
E	Ala634	Ala634	C
E	Arg635	Asp691, Thr692	C
E	Gln659	Glu719	C
E	Val662	Leu716	C
E	Arg928	Ser693	C
E	Phe931	Thr692,Ser693	C
E	Ala932	Ser693,Ile694	C
E	Gly934	Ile694,Met879	C
E	Asn935	Asn695-Asn699,Tyr880	C
E	Ala936	Asn877,Met879	C
E	Val937	Asn699, Arg701,Ser863	C
F*	Leu4	Asn60,Gln594	A*

ST2: H1—H4

A	Arg88	Tyr74,Pro75,Glu577	L
A	Arg312	Asn92,Gly94	L
A	Gly314	Ile71	L
A	Met315	Arg68	L
A	Glu547	Tyr74	L
A	Arg86	Thr718	K
A	Val303	Leu716,Thr718	K
A	Cys304	Thr718	K
A	Met315	Asp700	K
A	Val319	Pro705	K
A	Pro633	Thr629	K
A	Ala634	Asn631	K
A	Thr636	Thr692	K
A	Glu638	Thr692	K
A	Gln659	Leu716,Thr718	K
A	Ala932	Ser693	K
A	Thr933	Ser687,Leu689,Asn890	K
A	Asn935	Asn890	K
B*	Thr9	Gly698	K
B*	Arg14	Ile694	K
	Asp7	Lys65,Ala66	L*

ST3: H2—H3

D	Ile76	Ile76	G
D	Gln77	Tyr74,Ile76	G
D	Asp79	Arg72,Tyr74	G
D	Arg86	Tyr74	G
D	Arg312	Gly94,Asp95,Arg919	G
D	Gly314	Gly94	G
D	Met315	Lys65-Gln67,Trp97,His589	G
D	Glu547	Tyr74	G
D	Val937	Thr63,Glu64	G
D	Ser82	Thr718	I
D	Thr83	Thr718	I
D	Arg86	Glu719	I
D	Gly302	Pro705	I
D	Val303	Leu703-Pro705	I
D	Val319	Asp700	I
D	Pro322	Asn706	I
D	Lys615	Thr692,Ser693	I
D	Ile632	Thr692	I
D	Pro633	Thr692,Ser886	I
D	Arg635	Asp691-Ser693	I
D	Gln659	Glu719	I
D	Arg928	Ser693	I
D	Phe931	Thr692,Ser693	I
D	Ala932	Ser693,Ile694	I
D	Asn935	Ile694-Trp696,Met879,Tyr880	I
D	Ala936	Asn877,Met879	I
D	Val937	Asn699,Ser863,Leu874	I

(table continues in next page)

S12 Table (continued)

ST4: H4—H2

Chain	Residue	Interacting residues	Chain
L	Ile76	Ile76	E
	Gln77	Tyr74,Ile76	
	Thr78	Tyr74	
	Asp79	Arg72,Tyr74	
	Arg86	Tyr74	
	Arg88	Tyr74	
	Glu311	Arg919	
	Arg312	Gly94,Asp95,Arg919	
	Gly314	Gly94,Trp97	
	Met315	Lys65-Trp97,His589	
	Val319	Ala66	
	Val937	Thr62,Glu64	
	Ser82	Thr718	D
	Thr83	Thr718	
	Gly302	Pro705	
	Val303	Thr704,Pro705	
	Val319	Asp700	
	Pro322	Asn706	
	Lys615	Thr692	
	Ile632	Thr692	
	Pro633	Thr692,Ser886	
	Arg635	Asp691,Thr692	
	Gln659	Glu719	
	Arg928	Ser693	
	Phe931	Thr692,Ser693	
	Ala932	Ser693,Ile694	
	Thr933	Ser693,Ile694	
	Asn935	Ile694-Gly698	
	Ala936	Leu874,Asn877,Met879,Tyr880	

ST5: H3—H4

Chain	Residue	Interacting residues	Chain
I	Ile76	Ile76	J
	Gln77	Tyr74,Ile76	
	Thr78	Tyr74	
	Asp79	Arg72,Tyr74	
	Arg86	Tyr74	
	Arg312	Gly94,Asp95,Arg919	
	Ser313	Gly94	
	Gly314	Gly94,Trp97	
	Met315	Lys65,Ala66,Gln67,Trp97,His589	
	Val319	Ala66	
	Glu547	Tyr74	
	Val937	Glu64	
	Ser82	Thr718	L
	Thr83	Thr718	
	Gly302	Pro705,Asn706	
	Val303	Thr704,Pro705	
	Val319	Asp700	
	Pro322	Asn706	
	Lys615	Thr692,Ser693	
	Ile632	Thr692	
	Pro633	Thr692,Ser886	
	Arg635	Asp691,Thr692	
	Gln659	Glu719	
	Arg928	Ser693	
	Phe931	Thr692,Ser693	
	Ala932	Ile694	
	Thr933	Ser693,Ile694	
	Gly934	Met879	
	Asn935	Ile694,Trp696,Asn699,Met879,Tyr880	
	Ala936	Leu874,Asn877,Met879	
	Val937	Asn699,Ser863,Leu874,Asn877	

ST6: H3—H3 (AU1)

Chain	Residue	Interacting residues	Chain
H	Ile76	Ile76	I’
	Gln77	Tyr74,Ile76	
	Asp79	Arg72,Tyr74	
	Arg86	Tyr74	
	Glu311	Arg919	
	Arg312	Asp95,Arg919	
	Gly314	Gly94,Trp97	
	Met315	Lys65	
	Val319	Ala66	
	Asp691	Arg635	H’
	Thr692	Ile632,Pro633,Arg635,Phe931	
	Ser693	Lys615,Arg928,Phe931,Ala932,Thr933	
	Ile694	Ala932,Thr933,Gly934	
	Asp700	Val319	
	Thr704	Val303	
	Pro705	Gly302,Val303	
	Asn706	Gly302,Pro322	
	Leu716	Val662	
	Thr718	Thr81,Ser82	
	Glu719	Arg86,Gln659	
	Asn877	Asn935	
	Met879	Asn935	
	Tyr880	Asn935	
	Ser886	Pro633	

(table continues in next page)

S12 Table (continued)

ST7: H4—H3 (AU 1)			
J	Arg88	Tyr74	H’
J	Glu311	Asn92	H’
J	Arg312	Asn92	H’
J	Met315	Gln67,Arg68	H’
J	Glu547	Tyr74	H’
J	Arg86	Thr718	G’
J	Val303	Leu716,Thr718	G’
J	Cys304	Thr718	G’
J	Met315	Asp700	G’
J	Val319	Pro705	G’
J	Pro633	Thr629	G’
J	Ala634	Asn631	G’
J	Thr636	Thr692	G’
J	Phe927	Ser693	G’
J	Ala932	Ser693	G’
J	Thr933	Ser687,Leu689,Asn890	G’
K*	Asp7	Asn699	G’
K*	Thr9	Gly698,Asn699	G’
K*	Thr10	Asn699	G’
K*	Asp7	Ala66	H’*
K*	Thr9	Ala66	H’*

Interacting amino acids were identified with UCSF Chimera *findclash* [1] implemented in Scipion [2]. Following the nomenclature in [3], **ST**, **TT** and **SS** indicate the three kinds of interfaces between hexons (**S9 Figure, see also S13 and S14 Tables**). **S** refers to the facet of the hexon trimer pseudo-hexagonal base composed by the two β-barrels in a single monomer; **T** refers to the facet composed by two β-barrels coming from two different hexon monomers. **H1-H4** refer to the four hexon trimers in the icosahedral asymmetric unit. Suffixes _AU1 to _AU7 indicate neighbouring asymmetric units (**S9 Figure**). Letters **A-L** (blue shaded columns, see also **S9 Figure**) denote the 12 hexon monomer chains in the icosahedral asymmetric unit. A prime (’) symbol indicates chains belonging to the neighbouring asymmetric units. **Purple text** indicates residues potentially involved in salt bridges and underlining indicates recurrent salt bridges. Cells shaded in light orange indicate C-terminal flexible regions and pink shading indicates N-terminal flexible regions. These flexible regions were defined based on the RMSD analysis of the twelve hexon chains of the asymmetric unit (**S3d Fig.**). Residues involving difference regions of FAdV-C4 and HAdV-C5 (**S6 Table and Fig. 2a**), except those at the N- or -C termini, where not found in the interaction analysis, indicating that interactions between hexons are conserved between FAdV-C4 and HAdV-C5. Only the N- and -C termini, intrinsically flexible and variable, establish different interactions.

*Notice that some “S” interfaces, which are defined as involving a single hexon monomer on the basis of the hexagonal shape of the trimer, in fact may involve residues from two different monomers. This is due to the extensive interlacing of molecules in the hexon trimer, which results in the N-terminus of one hexon monomer reaching all the way to the center of the hexagon facet formed by the adjacent monomer [4].

References

1. Pettersen EF, Goddard TD, Huang CC, Couch GS, Greenblatt DM, Meng EC, et al. UCSF Chimera--a visualization system for exploratory research and analysis. J Comput Chem. 2004;25(13):1605-12. PubMed PMID: 15264254.

2. Martínez M, Jiménez-Moreno A, Maluenda D, Ramírez-Aportela E, Melero R, Cuervo A, et al. Integration of Cryo-EM Model Building Software in Scipion. J Chem Inf Model. 2020;60(5):2533-40. Epub 2020/01/30. doi: 10.1021/acs.jcim.9b01032. PubMed PMID: 31994878.

3. Liu H, Jin L, Koh SB, Atanasov I, Schein S, Wu L, et al. Atomic structure of human adenovirus by cryo-EM reveals interactions among protein networks. Science. 2010;329(5995):1038-43. Epub 2010/08/28. doi: 10.1126/science.1187433. PubMed PMID: 20798312; PubMed Central PMCID: PMCPMC3412078.

4. Rux JJ, Kuser PR, Burnett RM. Structural and phylogenetic analysis of adenovirus hexons by use of high-resolution x-ray crystallographic, molecular modeling, and sequence-based methods. J Virol. 2003;77(17):9553-66. Epub 2003/08/14. doi: 10.1128/jvi.77.17.9553-9566.2003. PubMed PMID: 12915569; PubMed Central PMCID: PMCPMC187380.
