# Supplementary material for: Aviadenovirus structure: A highly thermostable capsid in the absence of stabilizing proteins
Source: PLoS Pathog. 2025 Oct 9;21(10):e1013553. doi: 10.1371/journal.ppat.1013553 (PMC12517501; doi:10.1371/journal.ppat.1013553)
Supplement: S13 Table — (PDF) [file ppat.1013553.s014.pdf]

S13 Table. Interactions between hexons in different facets (TT interfaces). For interface nomenclature, see **S9 Figure.** Nomenclature and colour codes as in **S12 Table**.

Local two-fold axes

TT8: H1—H1 (AU 3)

C	Glu64<br>Lys65<br>Ala66	Lys65, Ala66<br>Glu64, Ala66<br>Ala66	B’
C	Lys65<br>Ala66<br>Arg68<br>Ile71<br>Asn90<br>Asn92<br>Asp95<br>Trp97<br>Arg312<br>Arg919	Asn699<br>Gly698-Asp700<br>Asn706<br>Ser693<br>Thr692<br>Asp691,Thr692,Ser693<br>Met879,Asn883<br>Ile694<br>Ala634<br>Asn883-His885	A’
B	Gly698<br>Asn699<br>Asp700	Asp700<br>Asp700<br>Asn706	A’
B	Asp691<br>Thr692<br>Ser693<br>Ile694<br>Met879<br>Asn883<br>Ser884<br>His885<br>Ser886	Asn92<br>Arg72,Phe73,Tyr74<br>Arg72,Tyr74<br>Arg68,Ile71<br>Gly94,Asp95,Trp97<br>Asn92-Gly94,Ser541<br>Glu311,Ser543<br>Asn92<br>Asn90	B’

TT9: H2- H4 (AU 3)

F	Glu64<br>Lys65<br>Ala66	Lys65, Ala66<br>Glu64, Ala66<br>Ala66	K’
E	Asp691<br>Thr692<br>Ser693<br>Ile694<br>Asn699<br>Asn883<br>Ser884	Asn92<br>Tyr74,Ile76<br>Arg72,Tyr74<br>Arg68<br>Ala66<br>Asp95, Arg919<br>Asn92, Glu311	K’
F	Lys65<br>Ala66<br>Ile71<br>Asn90<br>Asn92<br>Gly94<br>Asp95<br>Arg919	Asn699<br>Gly698-Asp700<br>Ser693<br>Thr692<br>Asp691,Thr692<br>Ile694<br>Asn883, His885<br>Ser884,His885	J’
E	Gly698<br>Asn699<br>Asp700	Asp700<br>Asp700<br>Pro705, Asn706	J’

Icosahedral 2-fold axis

TT10: H2—H2 (AU 5)

D	Arg68<br>Ile71<br>Arg72<br>Phe73<br>Tyr74<br>Ile76	Asn706<br>Ser693<br>Ser693<br>Thr692,Ser693<br>Leu689,Thr692,Asn890<br>Thr692	F’
F	Leu689<br>Thr692<br>Ser693<br>Asn706<br>Asn890	Tyr74<br>Phe73,Tyr74,Ile76<br>Ile71,Arg72,Phe73<br>Arg68<br>Tyr74	D’
F	Ala627<br>Gly698<br>Asp700<br>Leu703<br>Pro705<br>Asn706<br>Thr718	Thr718<br>Asp700<br>Gly698,Asp700<br>Asn706<br>Asn706<br>Leu703,Pro705<br>Ala627	F’

Note that TT_{10} has the lowest number of residues involved in interactions (28 residues), compared to TT_8 and TT_9 (with 43 and 33 residues interacting respectively). Moreover, TT_{10} does not have any pair of residues that could chemically form a salt bridge, thus the TT_{10} interaction is the weakest among the TT interactions. Interestingly, there is a remnant density (*RD4*) that could be strengthening the interaction (see text section “***Additional internal densities***”). The salt bridges at the local two-fold axes are conserved between genera [1]

Reference

1. Marabini R, Condezo GN, Krupovic M, Menéndez-Conejero R, Gómez-Blanco J, San Martín C. Near-atomic structure of an atadenovirus reveals a conserved capsid-binding motif and intergenera variations in cementing proteins. Sci Adv. 2021;7(14). Epub 2021/04/02. doi: 10.1126/sciadv.abe6008. PubMed PMID: 33789897; PubMed Central PMCID: PMCPMC8011978.
