# Supplementary material for: Aviadenovirus structure: A highly thermostable capsid in the absence of stabilizing proteins
Source: PLoS Pathog. 2025 Oct 9;21(10):e1013553. doi: 10.1371/journal.ppat.1013553 (PMC12517501; doi:10.1371/journal.ppat.1013553)
Supplement: S14 Table — (PDF) [file ppat.1013553.s015.pdf]

S14 Table. Interactions between hexons in different facets (SS interfaces). For interface nomenclature, see **S9 Figure.** Nomenclature and colour codes as in **S12 Table**.

Local two-fold axis									
**SS11: H4—H1 (AU 3)**					**SS12: H2—H3 (AU 5)**				
C	Asp79	Asn663		K’	F	Asp79	Asn663		G’
	Gly302	Val303,Gln659				Val303	Tyr660,Val662		
	Val303	Thr83,Gln659,Tyr660				Arg312	Ile630,Asn631,Ile632,Pro633		
	Arg312	Asn631,Ile632,Pro633				Met315	Asp323,Lys615		
	Ser313	Ile632				Val319	Val303		
	Met315	Asp323,Lys615				Glu320	Val303		
	Glu320	Val303				Leu321	Val303		
	Ala602	Ala936,Val937				Ala602	Ala936,Val937		
	Thr603	Val937				Asn631	Gln77		
	Pro633	Ile76,Gln77				Pro633	Ile76,Gln77,Arg88		
	Arg635	Arg88				Ala634	Arg88,Glu311		
	Thr636	Gly314				Thr636	Gly314		
	Gln659	Val662				Glu638	Met315		
	Asn882	Arg312				Gln659	Val662		
	Asn883	Arg312				Asn882	Glu311,Arg312		
	Ser884	Glu311				Asn883	Arg312		
	Asn915	Ala936				Ser884	Glu311		
	Glu918	Pro633,Ala634				Asn915	Ala936		
	Asn920	Pro633				Glu918	Ala634		
	Val921	Pro633				Asn920	Pro633		
	Thr933	Thr307				Val921	Pro633		
	Asn935	Asp79				Thr933	Thr307		
	Ala936	Arg86,Glu547				Asn935	Asp79,Arg86		
	Val937	Gln77				Ala936	Thr81		
A*	Arg14	Met315			D*	Arg14	Met315		

*Notice that some “S” interfaces, which are defined as involving a single hexon monomer on the basis of the hexagonal shape of the trimer, in fact may involve residues from two different monomers. This is due to the extensive interlacing of molecules in the hexon trimer, which results in the N-terminus of one hexon monomer reaching all the way to the center of the hexagon facet formed by the adjacent monomer [1].

Reference

1. Rux JJ, Kuser PR, Burnett RM. Structural and phylogenetic analysis of adenovirus hexons by use of high-resolution x-ray crystallographic, molecular modeling, and sequence-based methods. J Virol. 2003;77(17):9553-66. Epub 2003/08/14. doi: 10.1128/jvi.77.17.9553-9566.2003. PubMed PMID: 12915569; PubMed Central PMCID: PMCPMC187380.
