# Supplementary material for: Aviadenovirus structure: A highly thermostable capsid in the absence of stabilizing proteins
Source: PLoS Pathog. 2025 Oct 9;21(10):e1013553. doi: 10.1371/journal.ppat.1013553 (PMC12517501; doi:10.1371/journal.ppat.1013553)
Supplement: S15 Table — (PDF) [file ppat.1013553.s016.pdf]

S15 Table. Interactions between hexon and penton base.

H1-P				P-H1(AU 3)			
B	Thr83	Lys96	M	M	Asp71	Arg312	B’
	Arg86	Thr408			Tyr72	Glu311,Arg312	
	Val303	Ile91,Arg104			Thr73	Arg312	
	Ser305	Thr408					
	Thr307	Thr408					
	Gly314	Glu405					
	Met315	Glu405,Lys485					
	Asp323	Thr120					
	Glu547	Thr408					
	Pro633	Asp112,Leu113					
	Ala634	Arg70					
	Arg635	Arg70,Leu113					
	Thr636	Asp71					
	Gln655	Tyr94					
	Ala658	Tyr94					
	Gln659	Asn95,Lys96,Asp97					
	Tyr660	Lys96					
	Asp661	Tyr94			Arg56	Thr9,Thr10	C’*
	Asn663	Tyr94			Met58	Leu4,Thr5,Pro6	
	Ala932	Glu121					
	Thr933	Thr120,Ser122					
	Gly934	Ser122					
	Asn935	Tyr69,Ser122,Gln124					
C*	Leu8	Asn127					
	Thr9	Asn127,Arg128					

Nomenclature and colour codes as in the previous tables. **P** indicates the penton base monomer (with chain id M in the coordinate file). All hexon-penton interactions involve “S” facets in the hexon pseudohexagonal base (**S9 Figure**). Residues in regions differing from HAdV-C5 (***diff2* in S8 Table**) are shadowed in yellow. Notice that, although some penton residues involved in the interaction with hexon, are part of the difference regions, this is not the case for their partners in hexon. This observation indicates that hexon residues interacting with penton base are conserved, but penton interacting residues differ.

*Notice that some “S” interfaces, which are defined as involving a single hexon monomer on the basis of the hexagonal shape of the trimer, in fact may involve residues from two different monomers. This is due to the extensive interlacing of molecules in the hexon trimer, which results in the N-terminus of one hexon monomer reaching all the way to the center of the hexagon facet formed by the adjacent monomer.
