# Supplementary material for: Aviadenovirus structure: A highly thermostable capsid in the absence of stabilizing proteins
Source: PLoS Pathog. 2025 Oct 9;21(10):e1013553. doi: 10.1371/journal.ppat.1013553 (PMC12517501; doi:10.1371/journal.ppat.1013553)
Supplement: S16 Table — (PDF) [file ppat.1013553.s017.pdf]

S16 Table. Interactions between protein IIIa and other capsid components. Nomenclature and colour codes as in the previous tables.

IIIa interactions with hexon

IIIa-H1

	Domain			
N	VIII binding	Asp209	Arg26	A
		Ala210	Glu27	
		Val211	Arg26,Glu31	
	GOS-glue	Pro38	Gln876	B
		Tyr39	Gln876,Pro878	
		Ala40	Leu871,Gln876	
		Glu60	Asn601	
		Val66	Leu597	
		Lys67	Leu597	
		Pro77	Asn590,Asn593	
		Asp78	His589,Asn590,Asn593	
		Met80	Asn593,Gln594,Leu597	
		Gly81	Asn593,Glu596,Leu597	
		Ala82	Asp95,Arg919	
		His84	Leu597	
		Ser85	Glu918,Arg919	
		Leu88	Pro917,Glu918	
		Asn89	Arg312,Glu918	
	VIII-binding	Arg150	Arg26	
		Tyr237	Arg26,Glu31,Gln34,Gln35	
		Gly238	Arg26	
		Met240	Arg26	
		Pro242	Arg26	
	Core-binding	Arg251	Gln35	
		Lys254	Glu31	
	GOS-glue	Pro38	Val55,Ala56	C
		Ala40	Ala21	
		Asn41	Ala21,Gly22,Val55	
		Ile44	Pro13,Leu15,Gln16	
		Gln47	Pro13	
		Thr48	Pro13,Gln16	
		Val51	Ala11	
		Pro53	Ala11,Thr12	
		Lys54	Thr10	
		Asp56	Pro6	
	VIII-binding	Tyr142	Glu31	
		Lys143	Glu31,Gln35	
		Thr144	Glu31,Gln34,Gln35	
		Asp146	Arg26	

IIIa’ (AU3)-H1

	Domain			
N	GOS- glue	Tyr76	Arg59	C
		Asp78	Arg59	
		Arg90	Ala11,Pro13	
		Trp94	Thr10,Ala11	

(table continues in next page)

S16 Table (continued)

**IIIa interactions with penton base**			
**IIIa (AU3)-P**			
	Domain		
N	GOS-glue	Lys54 Arg56<br>Val55 Gln55,Arg56<br>Asp56 Arg56,Met58<br>Gly57 Gln55<br>Arg61 Gln55<br>Tyr92 Pro60,Thr61<br>Thr93 Thr61,Gly62<br>Trp94 Arg128	P
	Conecting helix	Asn95 Met58,Pro60<br>Gln100 Val57	

(table continues in next page)

S16 Table (continued)

IIIa interactions with other IIIa molecules					
**IIIa-IIIa'(AU3)**					
	Domain				*Domain*
N	GOS glue	Val18	Leu69	N'	GOS glue & connecting helix
		Ala19	Asp109		
		Ala21	Gln72		
		Leu22	Leu69,Gln72,Leu106,Asp109,Val110,Gly113		
		Ser23	Asp109,His112,Gly113		
		Ser24	Gln72,Gly113,Lys116		
		His25	Gln72,Lys116,Val117		
		Ala26	Gln72,Val117		
		Ala31	Gln72,Gly73,Ala74		
		Leu34	Ala74		
		Arg35	Gly73,Ala74,Tyr76		
		Tyr39	Gln79,Ile83		
		Arg42	Ala74,Ile75,Gln79		
		Leu43	Asp86		
		Leu46	Leu69,Ile75,Ile83		
		Gln47	Asp86,Arg90		
		Met50	Val65,Leu87,Val91,Ser102,Ile103,Leu106		
		Val51	Arg90,Val99		

(table continues in next page)

S16 Table (continued)

IIIa interactions with protein VIII (chain O)											
**IIIa-VIII**						**IIIa-VIII’ (AU3)**					
	Domain			*Domain*			*Domain*			*Domain*	
N	GOS glue	Glu71<br>Gly73<br>Tyr76<br>Pro77	Tyr234<br>Tyr234<br>Val233<br>Val233	Body	O	N	GOS glue	Ala26<br>Asn27<br>Arg35<br>Tyr36	Tyr234<br>Tyr234<br>Tyr234<br>Lys235	Body	O
	VIII binding	Asp236<br>Tyr237	Ser191,Gln192<br>Ser191	Neck							
