# Supplementary material for: Aviadenovirus structure: A highly thermostable capsid in the absence of stabilizing proteins
Source: PLoS Pathog. 2025 Oct 9;21(10):e1013553. doi: 10.1371/journal.ppat.1013553 (PMC12517501; doi:10.1371/journal.ppat.1013553)
Supplement: S17 Table — (PDF) [file ppat.1013553.s018.pdf]

S17 Table. Interactions between protein VIII (chain O) and hexons in the peripentonal region. For interactions between VIII and IIIa, see **S16 Table**. Nomenclature and colour codes as in the previous tables.

VIII-H1

Chain	*Domain*	VIII residue	Hexon residues	Hexon
O	Body	Asn16	Glu638,Asn882	A
		Val18	Glu638,Phe927	
		Thr19	Thr881	
		Ala23	Asn882	
		Asp88	Glu918	
		Val89	Leu597	
	Neck	Ser92	Leu597	
		Gly93	Asp95,Asn593	
		Pro94	Asn590,Asn593	
	Head	Leu112	Glu64,Lys65	
		Ser113	Lys65,Ala66	
		Gly114	Lys65	
		Glu175	Asn590	
		Thr177	Gln594	
	Neck	Phe181	Gln594	
		Lys182	Leu597	
		Leu185	Gln594,Met598, Asn601	
		Arg186	Leu597,Arg600, Asn601,Pro917	
		Val187	Asn601	
		Gln188	Asn601,Thr603	
		Gly189	Thr603	
		Pro190	Thr603	
	Body	Ala228	Asn882	
		Phe240	Asn877,Pro878,Met879	
		Glu241	Met879	
	Body	Pro17	Thr12,Pro13	B
		Val18	Pro13,Leu15	
		Gln73	Asp7	
		Pro75	Leu4,Thr5	
		Tyr76	Ala3,Leu4,Thr5	
		Ala77	Ala3,Thr5	
		Ile85	Ala3	
	Neck	Tyr184	Asp32	
		Leu185	Asp32	
		Val187	Ala2,Ala3	
		Gln188	Ser30,Asp32	
		Gly189	Ser30	
		Pro190	Tyr28	
		Ser191	Leu29,Glu31	
		Gln192	Glu27	
		Glu196	Pro23	
	Body	Val199	Gln16	
		Ser201	Thr12	
		Gln202	Thr12	
		Phe230	Arg59	
		Phe240	Thr58,Arg59	
		Glu241	Glu64	
	Head	Gly106	Met879,Asn883, His885	C
		Val107	His885	
		Arg109	Met879	
		Gln111	Gly698,Asn699	

VIII-H2

Chain	*Domain*	VIII residue	Hexon residues	Hexon
O	Neck	Gly93	Arg312	E
		Pro95	Arg312,Glu918,Val921	
		Ser97	Pro917,Glu918	
		Ala98	Asn915,Pro917	
		Val99	Ala602,Asn915	
		Pro101	Ala925	
	Head	Gln108	Arg635,Thr636, Glu638,Phe927, Thr929,Ala932	
		Arg109	Ala932,Gly934,Ala936	
		Val110	Tyr926,Phe927	
		Gln111	Val319,Tyr926,Thr933	
		Ser113	Asn316,Val317,Met924	
		Gly114	Ala923,Met924	
		Gly115	Ser313,Met315,Leu922	
	Neck	Tyr100	Asp7	F
		Pro101	Arg14,Tyr17	
	Head	Asp103	Arg14	
		Val110	Phe18	
		Pro174	Pro6,Asp7	
		Glu175	Leu4,Thr5,Pro6	
		Met176	Leu4,Thr5,Asp7	
		Thr177	Ala3	
		Pro178	Ala3,Thr5	
	Neck	Phe181	Ala2	

(table continues in next page)

S17 Table (continued)

VIII-H4				
	Domain			
O	Body	Ala7	Val937	K
		Pro8	Val937	
		Val12	Asn882,Ser884	
		Trp13	Asn883,Ser884	
		Lys14	Ser884	
		Pro17	Asp691,Ile694	
		Val18	Ser693	
		Gln26	Ser884	
		Asn28	Thr636	
		Tyr29	Ala634,Arg635,Ala932	
		Gly30	Thr636,Thr929, Pro930,Ala932	
		Ala31	Thr636,Ala932	
		Thr32	Phe927,Ala932	
		Ile33	Ala925,Phe927, Gly934,Asn935	
		Asp34	Ala925,Tyr926,Gly934	
		Trp35	Asn935,Val937	
		Val36	Asn935	
		Leu37	Met315,Asn316	
		Pro38	Met315,Asn316, Val317,Val319, Ala923,Met924	
		Gly39	Met315,Ala923,Met924	
		Gly40	Met315	
		Ser42	Pro917,Ala923	
		Phe43	Arg913,Asn915, Ala923-Ala925	
		Arg51	Val937	
		Thr60	Asn882	
		Phe67	Asn877,Pro878	
		Gln202	Met879	
		Met206	Pro878,Met879	
		Pro211	Asn882	
		Ala31	Arg14	L
		Ile33	Phe18	
		Phe43	Tyr17	
		Ala46	Tyr17	
		Ile50	Arg14,Tyr17	
		Arg53	Pro13	
		Phe67	Arg59	
		Glu70	Arg59,Asn60	
		Ser71	Arg59,Glu64	
		Asp72	Thr63,Asp588,Asn590	
		Gln73	Lys65	
		His80	Asp95,Asn593	
		Glu81	Lys65	
		Ile84	Asp95	

VIII (AU3)-H1				
	Domain			
O	Body	Met1	Pro13,Gln16	A
		Asn2	Gln16	
		Leu3	Pro13,Gln16,Tyr17	
		Leu4	Pro23,Tyr28	
		Val36	Pro13,Arg14,Tyr17	
		Leu37	Phe18	
		Ala44	Pro13	
		Phe218	Ser30,Asp32	
		Asp219	Glu31	
		Tyr11	Asn601	C
		Lys14	Glu918	
		Gln26	Glu918	
		Gln27	Asn601,Ala602,Pro917	
		Tyr29	Pro917,Glu918,Val921	
		Leu37	Phe927	
		Phe218	Met598,Asn601	
		Asp224	Leu597	
		Ala225	Leu597	
		Pro227	Leu597	
		Lys235	Asn60	
		Gly236	Gln594	
		Thr237	Asn590	
		Asn238	Asn590,Asn593,Gln594,Leu597	
		Ala239	Asn590	
		Glu241	Lys65,His589	
