# Supplementary material for: Aviadenovirus structure: A highly thermostable capsid in the absence of stabilizing proteins
Source: PLoS Pathog. 2025 Oct 9;21(10):e1013553. doi: 10.1371/journal.ppat.1013553 (PMC12517501; doi:10.1371/journal.ppat.1013553)
Supplement: S18 Table — (PDF) [file ppat.1013553.s019.pdf]

S18 Table. Interactions between protein VIII (chain P) and hexons in the central plate region. Nomenclature and colour codes as in the previous tables.

VIII-H4

	Domain			
P	Body	Asn16	Glu638,Asn882	J
		Val18	Glu638,Phe927	
		Ala23	Asn882	
		Ile84	Glu918	
		Asp88	Glu918	
		Val89	Leu597	
	Neck	Gly93	Asn593	
		Pro94	Asn590,Asn593	
	Head	Leu112	Lys65,Ala66	
		Ser113	Ala66	
		Gly114	Lys65	
		Gly115	Lys65	
		Glu175	Asn590	
		Met176	Asn590	
		Thr177	Asn590,Gln594	
	Neck	Lys182	Leu597	
		Leu185	Gln594,Met598	
		Arg186	Leu597,Arg600, Asn601,Pro917	
		Gln188	Asn601,Thr603	
		Gly189	Thr603	
		Pro190	Thr603	
	Body	Ala228	Pro878,Asn882	
		Phe240	Asn877,Pro878,Met879	
		Glu241	Met879	
	Body	Tyr15	Thr12	K
		Pro17	Thr12,Pro13	
		Val18	Arg14,Leu15	
		Thr19	Leu15	
		Gln73	Asp7	
		Pro75	Thr5	
		Tyr76	Ala3,Leu4	
		Ala77	Ala3,Thr5	
		Ile85	Ala3,Thr5	
		Tyr183	Ala2	
		Leu185	Asp32	
		Val187	Ala2,Ala3	
		Gln188	Glu31,Asp32	
		Gly189	Leu4,Ser30	
		Pro190	Leu4,Tyr28	
		Ser191	Glu31	
		Gln192	Pro23,Glu27	
		Glu196	Gln16	
	Neck	Val199	Gln16	
	Body	Ser201	Thr12	
		Gln202	Thr12	
		Phe230	Arg59	
		Phe240	Thr58,Arg59,Glu64	
	Head	Gly106	Met879,Asn883,His885	L
		Val107	Asn883,His885	
		Arg109	Met879	
		Gln111	Gly698,Asn699	

VIII-H3

	Domain			
P	Neck	Arg91	Arg312	I
		Gly93	Arg312	
		Pro95	Glu918,Val921	
		Ser97	Pro917,Glu918	
		Ala98	Asn915,Pro917	
		Val99	Ala602,Asn915	
		Pro101	Ala925	
	Head	Gln108	Thr636,Glu638, Phe927,Thr929,Ala932	
		Arg109	Ala932,Gly934,Ala936	
		Val110	Tyr926,Phe927,Gly934	
		Gln111	Val319,Tyr926	
		Leu112	Ala925	
		Ser113	Val317,Val319,Met924,Tyr926	
		Gly114	Val317,Ala923,Met924	
		Gly115	Ser313,Met315,Leu922,Ala923	
	Head	Pro101	Tyr17	G
		Phe102	Leu8	
		Gln108	Arg14	
		Val110	Phe18	

(table continues in next page)

S18 Table (continued)

VIII-H3 (AU1)				
	Domain			
P	Body	Ala6	Val937	G
		Pro8	Val937	
		Thr9	Val937	
		Val12	Asn882,Ser884	
		Trp13	Asn883,Ser884	
		Lys14	Ala634,Ser884	
		Pro17	Asp691,Ile694	
		Val18	Ser693	
		Gln26	Ala634,Ser884	
		Asn28	Thr636	
		Tyr29	Ala634,Arg635,Ala932	
		Gly30	Arg635,Thr636, Thr929,Pro930,Ala932	
		Ala31	Thr636,Glu638,Ala932	
		Thr32	Phe927,Ala932, Gly934,Asn935	
		Ile33	Ala925,Tyr926,Asn935	
		Asp34	Ala925,Tyr926,Gly934	
		Trp35	Asn935,Val937	
		Val36	Asn935	
		Leu37	Met315	
		Pro38	Asn316,Val317,Met924	
		Gly39	Ala923,Met924	
		Ser42	Ala923	
		Phe43	Arg913,Asn915, Ala923-Ala925	
		Thr60	Asn882	
		Phe67	Asn877,Pro878	
		Gln202	Met879	
		Phe205	Asn883	
		Met206	Pro878,Met879,Asn883	
		Pro211	Asn882	
		Ala31	Arg14	H
		Ile33	Arg14,Phe18	
		Phe43	Tyr17	
		Ala46	Tyr17	
		Ile50	Arg14,Tyr17	
		Arg51	Arg14	
		Arg53	Pro13	
		Phe67	Thr58,Arg59	
		Glu70	Arg59	
		Ser71	Arg59,Glu64	
		Asp72	Thr63,Glu64,Lys65, Asp588,Asn590	
		His80	Asp95,His589	
		Glu81	Lys65	
		Ile84	Asp95	

VIII (AU3)-H2				
	Domain			
P	Body	Asn2	Gln16	D
		Leu3	Pro13,Gln16,Tyr17	
		Leu4	Pro23,Glu27,Tyr28	
		Trp35	Pro13	
		Val36	Arg14,Tyr17	
		Leu37	Phe18	
		Gly40	Arg14	
		Ala44	Pro13	
		Phe218	Ser30,Asp32,Glu31	
		Asp219	Asp32	
		Tyr11	Asn601	F
		Lys14	Glu918	
		Gln26	Glu918	
		Gln27	Asn601	
		Tyr29	Pro917,Val921	
		Leu37	Tyr926,Phe927	
		Pro217	Asn601	
		Phe218	Leu597,Met598,Asn601	
		Asp224	Leu597	
		Ala225	Leu597	
		Pro227	Leu597	
		Gly236	Gln594	
		Thr237	Asn590	
		Asn238	Asn590,Asn593,Gln594	
		Ala239	Asn590	
		Glu241	Lys65,His589,Asn590	

Nomenclature and colour codes as in the previous tables.
