# Supplementary figures and images for: Aviadenovirus structure: A highly thermostable capsid in the absence of stabilizing proteins
Source: PLoS Pathog. 2025 Oct 9;21(10):e1013553. doi: 10.1371/journal.ppat.1013553 (PMC12517501; doi:10.1371/journal.ppat.1013553)

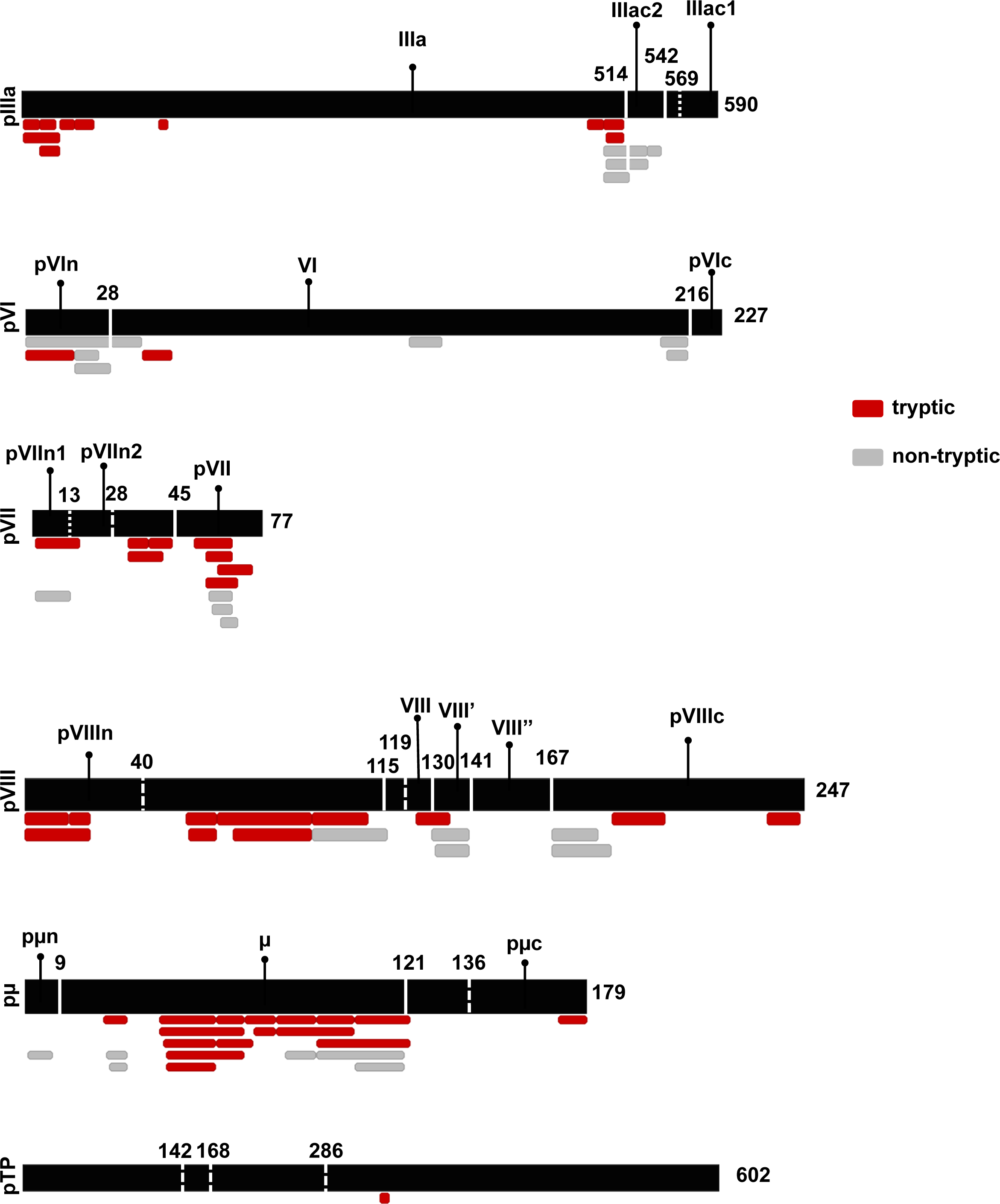

Supplement: S1 Fig — Total lengths of immature polypeptides (in amino acids) are indicated at the right hand side. The MASCOT search was adjusted for tryptic or non-tryptic small peptides. Red bars represent fragments excised by trypsin (cuts at C-termini of Lys and Arg, except if followed by Pro). Grey bars represent non-tryptic detected fragments, presumed to result from the endogenous AVP action. Continuous white lines indicate predicted canonical AVP sites experimentally observed in the LC-MS/MS assays. Dashed white lines indicate predicted canonical AVP sites, but not experimentally observed. Canonical AVP target sites are [MIL]xGx|G and [MIL]xGG|x [45]. Dotted lines indicate non-canonical AVP sites reported in the literature for other AdVs. Prefix “p” indicates precursor; “n”, N-terminal excised peptide; “c”, C-terminal excised peptide. L1-52/55k, which in HAdV-C5 is cleaved at multiple non-canonical sites [85], is not included here due to its complexity. The detection of peptides ending at predicted AVP cleavage sites helps confirm the universality of the AVP mechanism across the members of the Adenoviridae family and demonstrates that excised peptides are retained inside the capsid and not removed during assembly. Note that our structure indicates that the predicted cleavage at residue 40 in protein VIII does not occur, since we were able to continuously trace the polypeptide without interruption in that zone. Also, the central zone in pVIII is predicted to be cleaved at five sites, of which four can be confirmed in our LC-MS/MS assay but are not traced in our structure. The central region of pVIII in HAdV-C5 is cleaved at three sites [45]. Differences in the cleaved sites could be significant for capsid stability, as proposed for HAdV-F41 [38]. (PNG) [file ppat.1013553.s020.png]

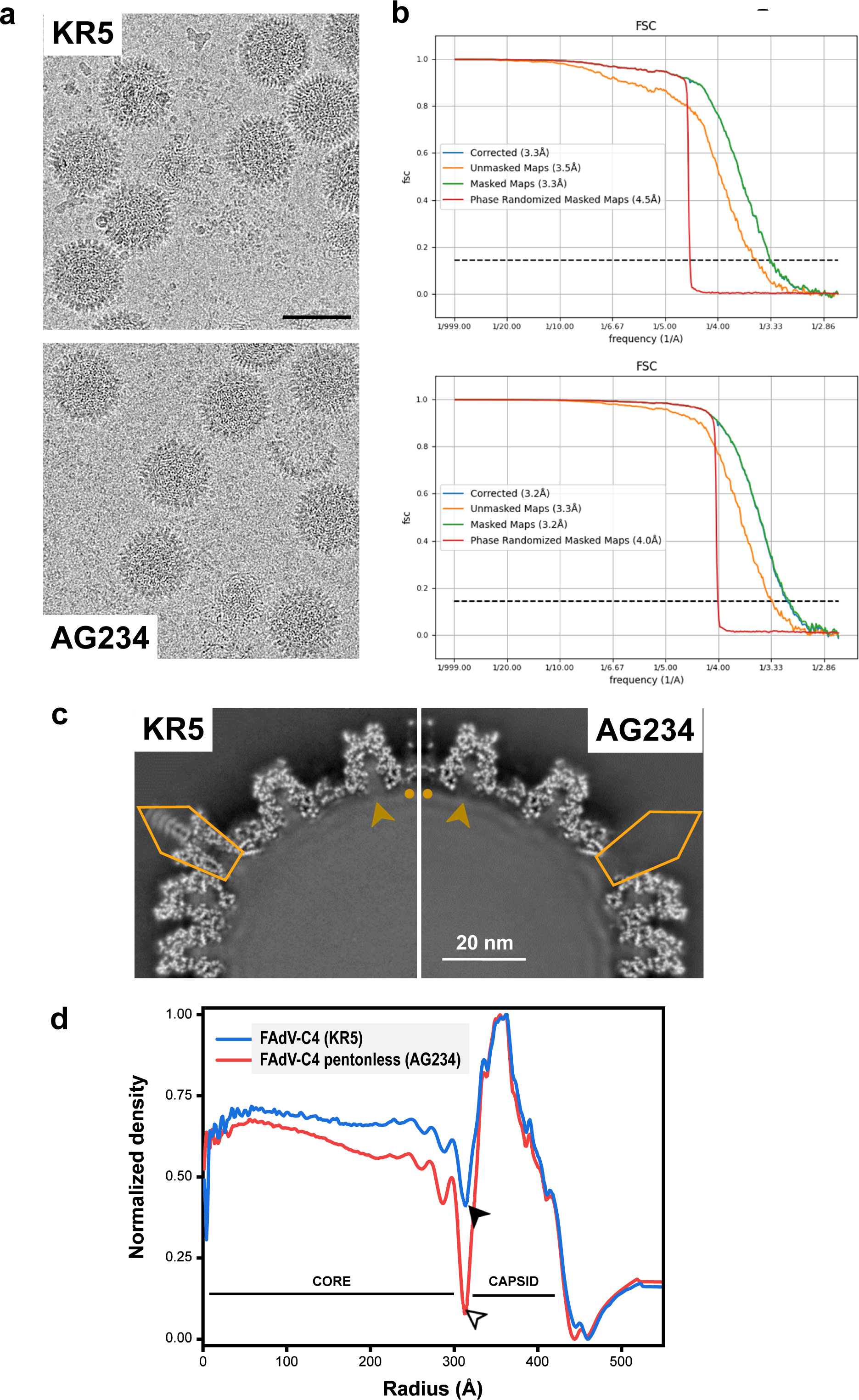

Supplement: S2 Fig — (a) Representative area of motion-corrected micrographs for the two specimens used (FAdV-C4 strains KR5 and AG234, as indicated). The bar represents 100 nm. (b) Fourier Shell Correlation (FSC) curves, as provided by RELION postprocess. Note that the corrected FSC curves (blue) overlap with the curve for the masked maps (green). Resolution values at the intersection of the FSC curves with the FSC = 0.143 threshold (dotted black line) are indicated in the plot legends. (c) A quadrant of the central slice of the density maps viewed along a 2-fold icosahedral axis. Notice the lack of pentons in AG234. Pentagons: presence or absence of pentons. Arrowheads: presence or absence of capsid-core connections. Dots: presence or absence of RD4 (see Fig 5). (d) Radial average profiles of the FAdV-C4 (KR5) and FAdV-C4 pentonless (AG234) maps. The filled arrowhead indicates the presence of density in regions connecting capsid and core, while the hollow arrowhead points to a large dip for the pentonless map, indicating loss of capsid-core connections. (PNG) [file ppat.1013553.s021.png]

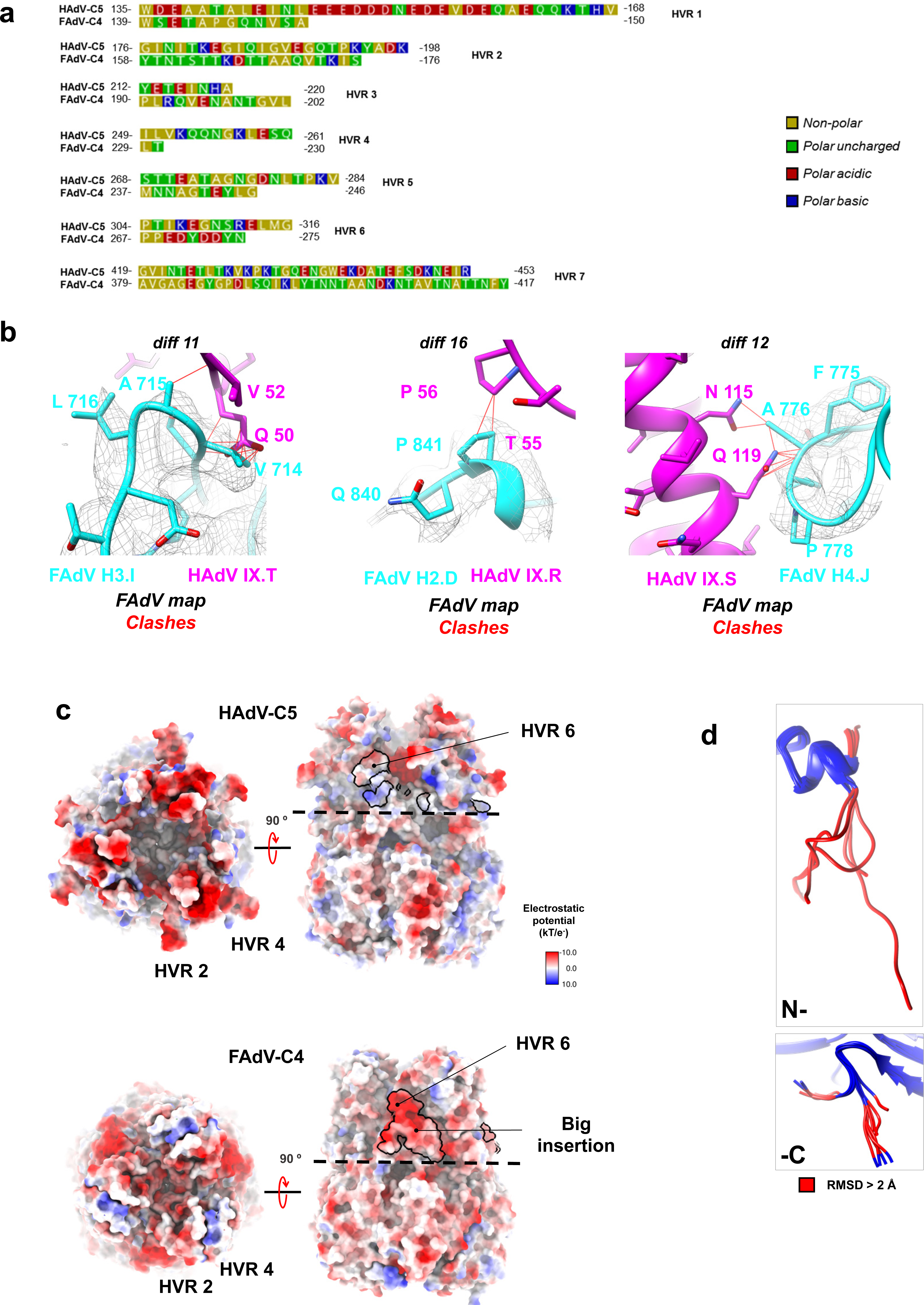

Supplement: S3 Fig — (a) Sequence alignment for HAdV-C5 and FAdV-C4 hexon, focusing on the hypervariable regions HVR1 to HVR7. (b) Residues in the pincer region in relation to the position of protein IX in HAdV-C5. Three diff regions of the FAdV-C4 hexon are depicted in cyan and with the density map in grey mesh. HAdV-C5 protein IX is in pink (PDB ID: 6b1t). Hexon number (H2-4) and chain IDs are indicated. Clashes are indicated with red lines. (c) HAdV-C5 (PDB ID 6cgv) and FAdV-C4 hexon trimer surfaces coloured by electrostatic potential. Note that the shorter HVR2 and HVR4 in FAdV-C4 result in more compact hexon towers when compared to HAdV-C5. The big insertion (diff 14) and its neighbourhood (diff 10 and 15) are highlighted in black, and contribute to a negatively charged patch in FAdV-C4. (d) Focus on the hexon termini after superposition of the twelve hexon monomers in the AU of FAdV-C4, coloured by RMSD with conserved regions in blue and residues exceeding 2 Å RMSD in red. (PNG) [file ppat.1013553.s022.png]

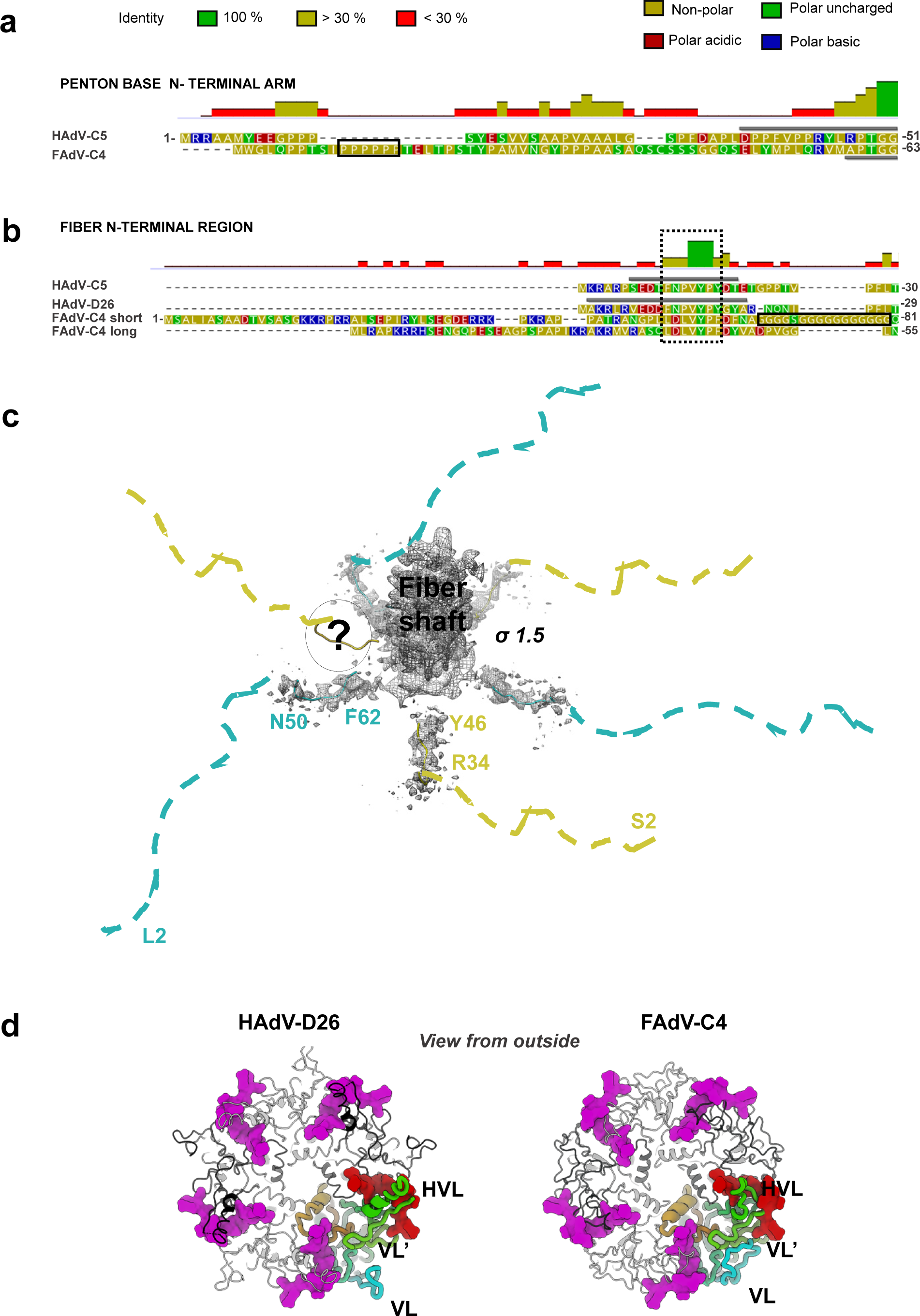

Supplement: S4 Fig — (a) Sequence alignment for HAdV-C5 and FAdV-C4 penton base, focusing on the N-terminal arm. The black rectangle highlights a poly-proline stretch. (b) Sequence alignments for the HAdV-C5, HAdV-D26, long and short FAdV-C4 fibres, focusing on the N-terminal peptides. A black rectangle highlights the poly-glycine stretch in the FAdV-C4 short fibre. The conserved penton binding motives are indicated with a dashed rectangle. In both (a) and (b), grey bars indicate regions traced (PDB ID:6b1t for HAdV-C5 penton base, 3izo and 5tx1 for HAdV-C5 and HAdV-D26 fibres), the histograms above the sequences indicate the mean pairwise identity over all pairs in the column, and amino acids are coloured by polarity according to the colour legends at the top. (c) Icosahedrally averaged density for fibre(s) in FAdV-C4 (map threshold = 1.5σ), with the penton binding peptides of the long (cyan) and short (yellow) fibres fitted. The question mark indicates lack of density for one of the six N-terminal tails. There is no density to accommodate the 30 (long fibre) or 50 (short fibre) amino acids preceding the conserved penton binding motif (dashed lines). (d) Top view of the HAdV-D26 and FAdV-C4 penton base pentamers with the fibre N-terminal peptide modeled for HAdV-D26 overlaid. The positions of the HVL, VL and VL’ are indicated for one penton base monomer (rainbow coloured). One fibre peptide is depicted in red, the other four occupying the rest of possible binding sites are in pink. (PNG) [file ppat.1013553.s023.png]

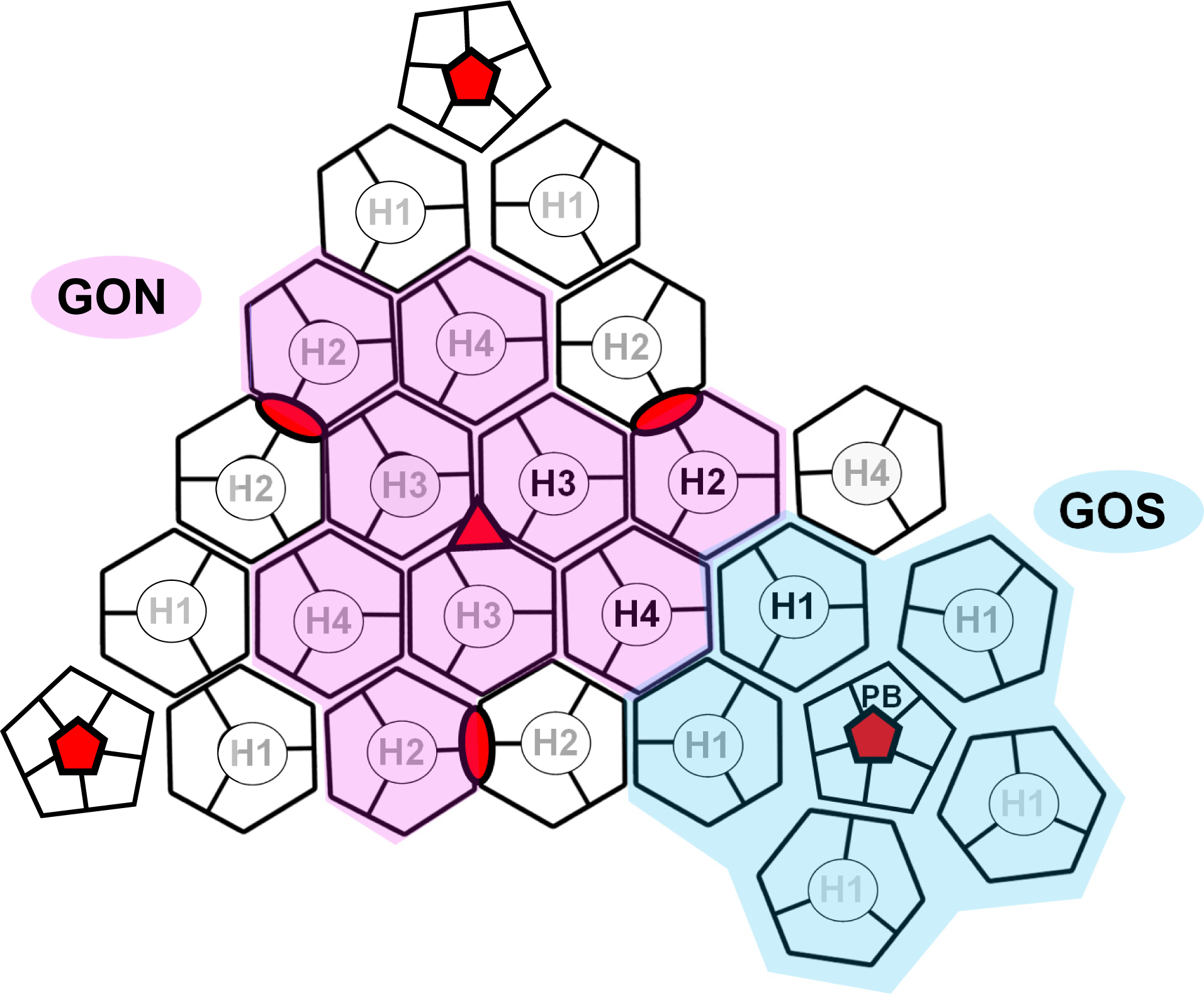

Supplement: S5 Fig — The four hexons (H1-H4) and one penton base monomer (PB) forming the icosahedral AU are labelled in black. Other hexons in the same or adjacent facets are labelled in grey. The view is from outside the capsid. Icosahedral symmetry axes are indicated with red symbols. The capsid organization can be described by two sets of tiles: (1) the Group of Six (GOS, cyan), containing one penton base pentamer and its five surrounding hexon trimers; and (2) the Group of Nine (GON, pink), containing the nine hexon trimers forming the central plate of each facet. (PNG) [file ppat.1013553.s024.png]

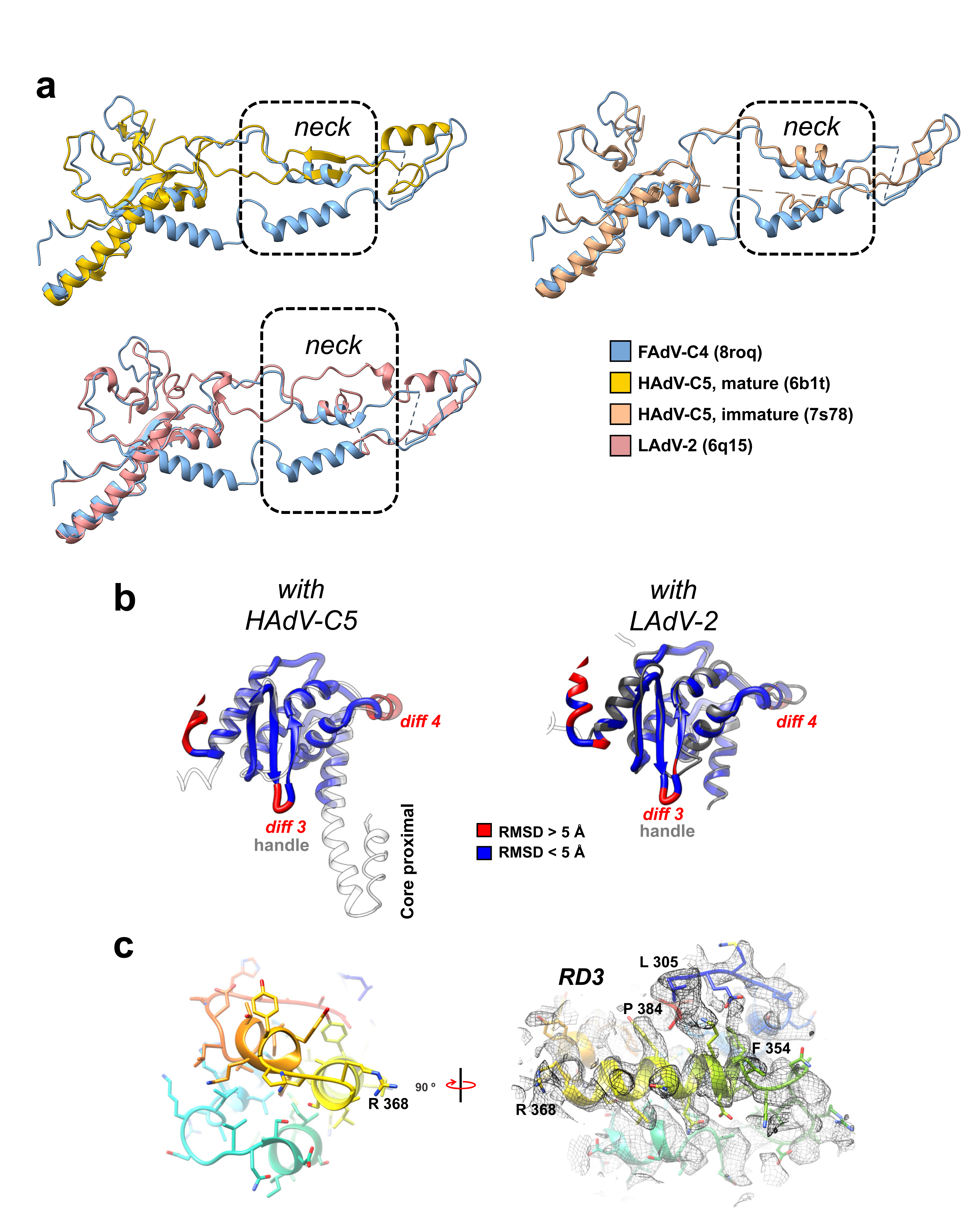

Supplement: S6 Fig — (a) Superposition of the FAdV-C4 protein VIII onto those of mature and immature HAdV-C5 (top), and the atadenovirus LAdV-2 (bottom). PDB IDs are indicated in the color legend. The dashed rectangle highlights the neck domain. (b) Superposition of the VIII-binding domains of HAdV-C5 (left, white) and LAdV-2 (right, grey) with the FAdV-C4 protein, oriented as in Fig 4a and 4b and coloured by RMSD. (c) Interpretation of remnant density RD3. Left: Rainbow-coloured APD domain of IIIa tentatively traced as a four α-helix bundle comprising amino acids 305–384. Right: 90º rotated model, with the density map. (PNG) [file ppat.1013553.s025.png]

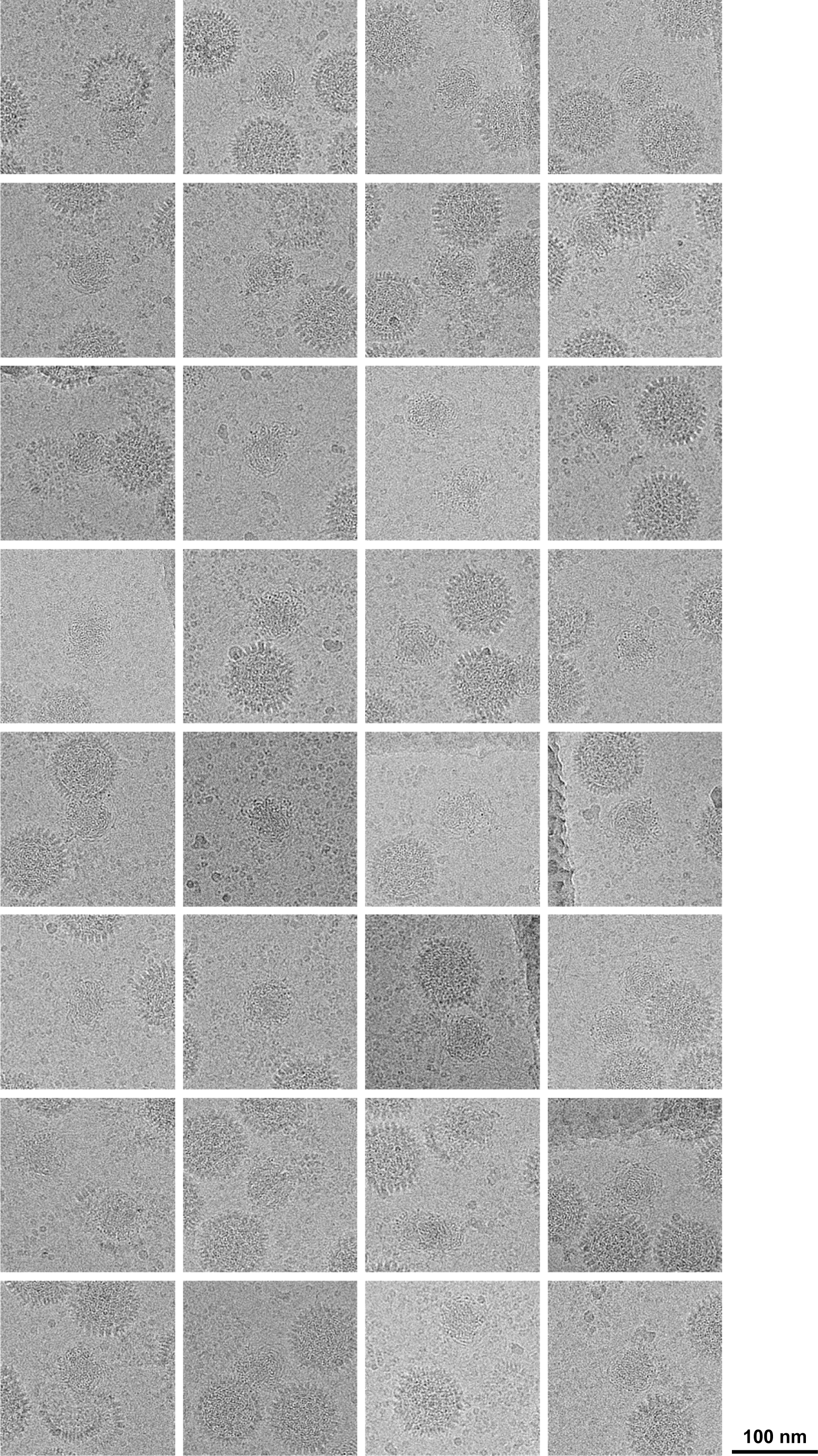

Supplement: S7 Fig — Gallery of cryo-EM images showing cores released from FAdV-C4 particles (AG243). (PNG) [file ppat.1013553.s026.png]

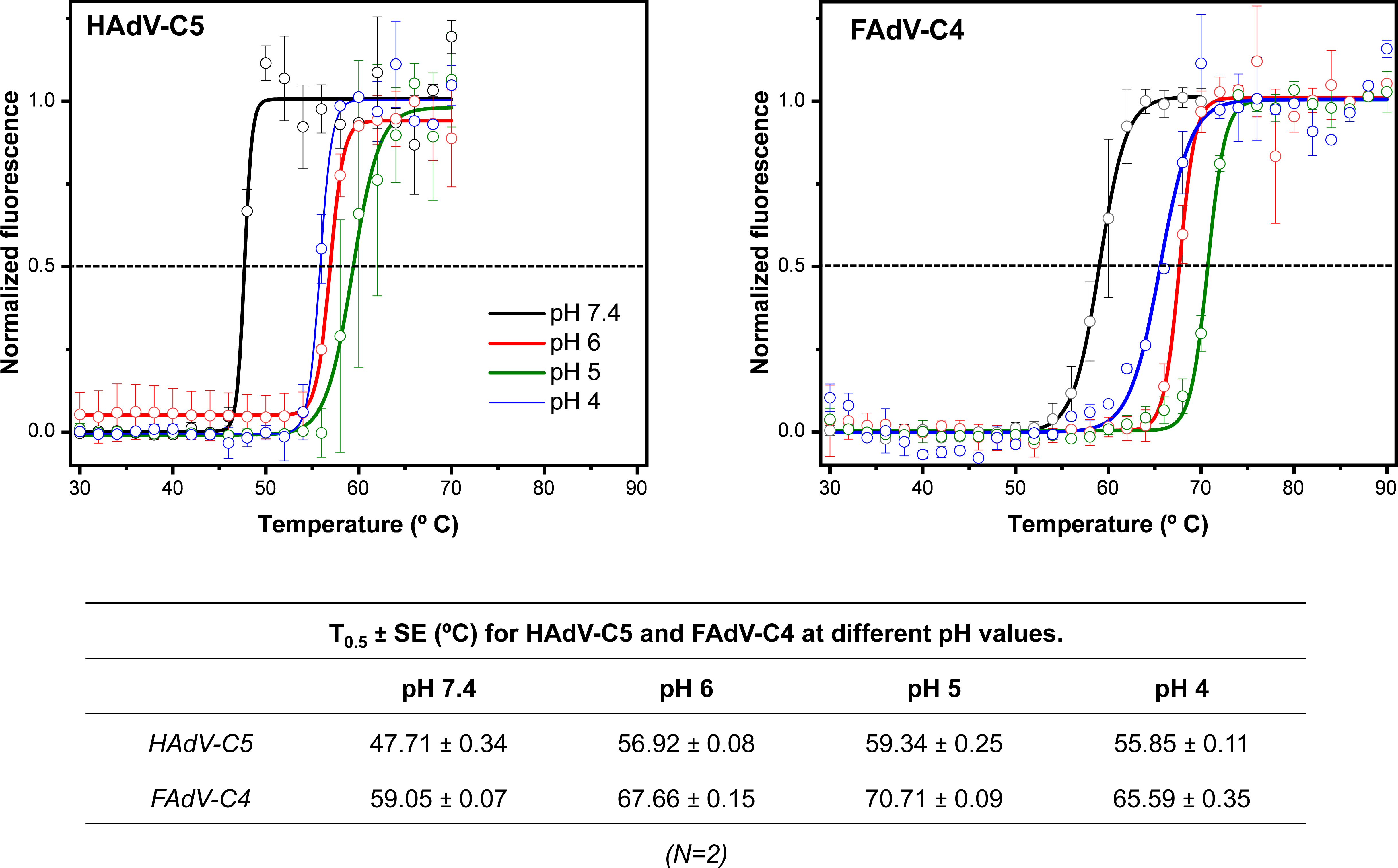

Supplement: S8 Fig — Open circles represent the normalized fluorescence ± STD. Continuous lines correspond to the Boltzmann sigmoid fitting to estimate the T0.5 for each condition tested (shown in the table at the bottom). Note the increased stability of FAdV-C4 for all the conditions, compared to HAdV-C5. (PNG) [file ppat.1013553.s027.png]

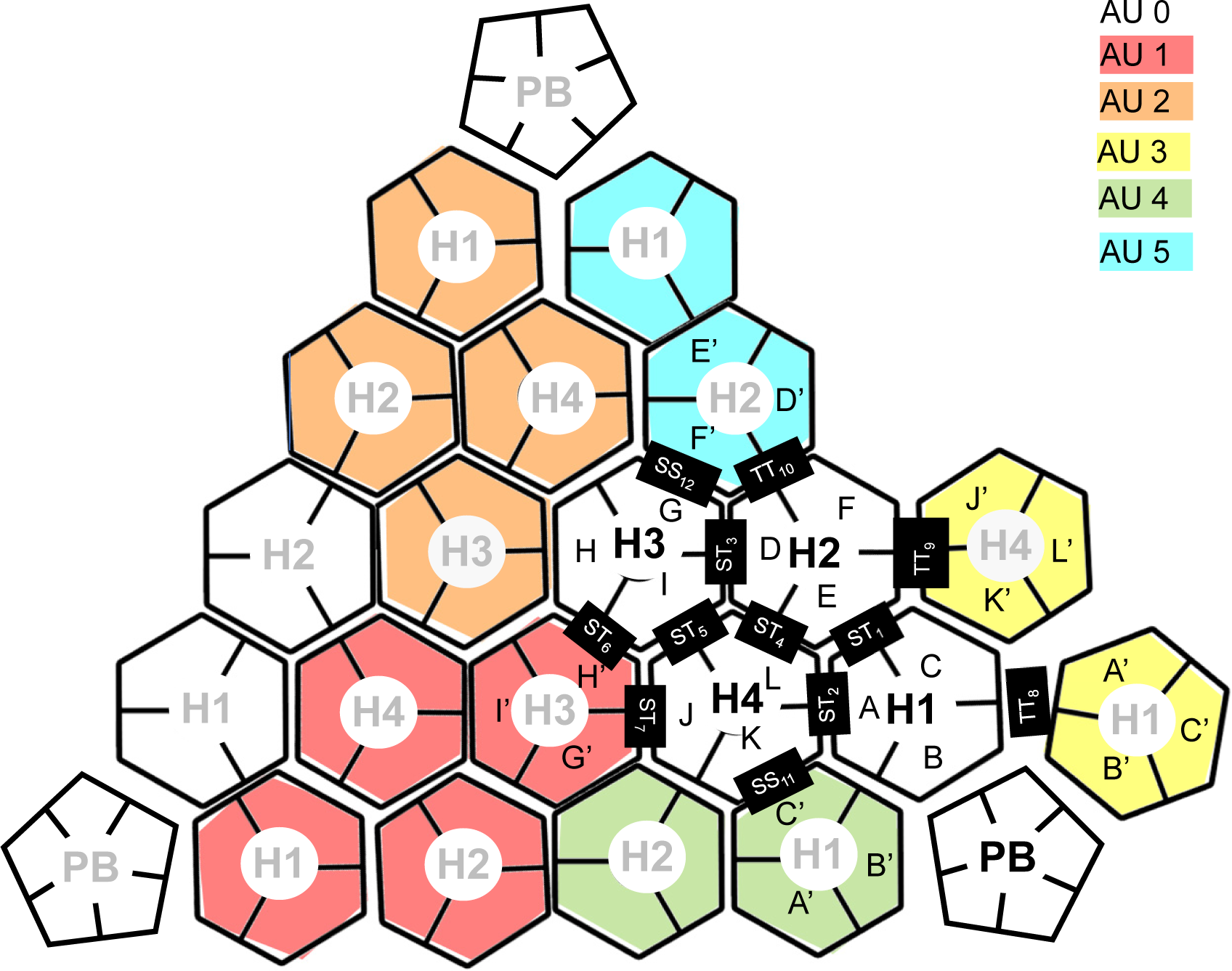

Supplement: S9 Fig — Hexons 1–4 in one AU (AU0) are depicted in white and labelled with black text. Those in neighbouring AUs (AU1-AU5) are coloured according to the colour key at the upper right corner and labelled with grey text. Letters A-L identify different hexon chains. Interfaces between hexons are indicated in black rectangles, with S designating the facet of the hexon pseudo-hexagonal base formed by the two β-barrels in a single monomer, and T indicating the facet of the hexon pseudo-hexagonal base formed by two β-barrels belonging to two adjacent hexon monomers [3]. This figure accompanies the interaction data shown in S12 to S18 Tables. (PNG) [file ppat.1013553.s028.png]
